# HSP90 inhibitor 17-DMAG exerts anticancer effects against gastric cancer cells principally by altering oxidant-antioxidant balance

**DOI:** 10.18632/oncotarget.17007

**Published:** 2017-04-10

**Authors:** Jeong Goo Kim, Sang Chul Lee, Ok-Hee Kim, Kee-Hwan Kim, Kyo Young Song, Sang Kuon Lee, Byung Jo Choi, Wonjun Jeong, Say-June Kim

**Affiliations:** ^1^ Department of Surgery, Daejeon St. Mary's Hospital, College of Medicine, The Catholic University of Korea, Seoul, Republic of Korea; ^2^ Department of Surgery, Seoul St. Mary's Hospital, College of Medicine, The Catholic University of Korea, Seoul, Republic of Korea

**Keywords:** 17-DMAG, antioxidant enzymes, HSP90 inhibitor, reactive oxygen species, stomach cancer

## Abstract

Heat shock protein 90 (HSP90) stabilizes numerous oncoproteins and, therefore, its inhibition has emerged as a promising antineoplastic strategy for diverse malignancies. In this study, we determined the therapeutic effects and mechanisms of action of a specific HSP90 inhibitor, 17-dimethylamino-ethylamino-17-demethoxygeldanamycin (17-DMAG), in gastric cancer cell lines (AGS, SNU-1, and KATO-III), patient-derived tissues, and a mouse xenograft model. 17-DMAG exerted anticancer effects against gastric cancer cells, manifested by significantly decreased proliferation rates (*P* < 0.05) and increased expression of apoptotic markers. Flow cytometry using dichlorofluorescein (DCF) diacetate revealed that 17-DMAG dose-dependently increases reactive oxygen species (ROS) levels in gastric cancer cells. Inhibition of ROS by N-acetyl-L-cysteine (NAC) abrogated the proapoptotic effects of 17-DMAG, as demonstrated by the decreased expression of proapoptotic proteins. In addition, 17-DMAG dose- and time-dependently reduced the expression of antioxidants such as catalase and glutathione peroxidase (GPx). Moreover, 17-DMAG reduced the expression of nuclear respiratory factor (NRF)-1 and NRF-2, and prevented them from migrating from the cytoplasm to the nucleus dose-dependently. Finally, in a nude mouse xenograft model, the shrinkage of tumors was more prominent in mice treated with 17-DMAG than in control mice (*P* < 0.05). Taken altogether, our results suggest that 17-DMAG exerts potent antineoplastic activity against gastric cancer cells primarily by promoting ROS generation and suppressing antioxidant enzyme activities.

## INTRODUCTION

Stomach cancer is the third leading cause of cancer-related deaths worldwide, with 1,000,000 new cases diagnosed each year [1]. Although systemic chemotherapy is a cornerstone in the treatment of advanced gastric cancer, survival rates after treatment remain discouraging [2]. Currently, heat shock protein 90 (HSP90) inhibitors have been the focus of attention because of their novel method of inhibiting tumor cell survival. HSP90 is an adenosine triphosphate (ATP)-dependent molecular chaperone, which assists and stabilizes the structural conformation of its client proteins by folding or assembly. Increased expression of HSP90 has been reported in a range of solid tumors originating from the esophagus, lung, breast, and pancreas [3]. Studies have revealed that a higher expression level of HSP90 correlates with clinicopathologic characteristics and prognosis in numerous cancers [4–6]. Because HSP90 client proteins include a number of oncogenic proteins, targeting HSP90 using specific inhibitors could be a promising anticancer strategy [7]. Moreover, HSP90 inhibitors have several outstanding advantages over other chemotherapeutic regimens. First, they target numerous oncoproteins rather than single one. In addition, they have the potential to increase tumor cell vulnerability to chemotherapeutic drugs [8]. Clinically, HSP90 inhibitors have exhibited encouraging results in a variety of cancers including melanoma, acute myeloid leukemia, castrate-refractory prostate cancer, non-small cell lung carcinoma, and multiple myeloma [9].

Of the various HSP90 inhibitors, geldanamycin (GA), a benzoquinone ansamycin compound, belongs to the originally identified class. Subsequently, derivatives of GA have been developed such as the less toxic 17-allylamino-17-demethoxygeldanamycin (17-AAG) as well as 17-dimethylamino-ethylamino-17-demethoxydeldanamycin (17-DMAG), which is water-soluble and, therefore, orally available. In addition to its enhanced oral bioavailability, 17-DMAG has numerous advantages over 17-AAG, including less hepatotoxicity, higher potency, less extensive metabolism, and a longer plasma half-time [9, 10]. A phase I trial of 17-DMAG validated its clinical usefulness in various cancer types including acute myeloid leukemia (partial response), castration-refractory prostate cancer (complete response), melanoma (partial response), renal cancer, and chondrosarcoma (stable disease) [11]. However, to the best of our knowledge, there have been no reports of the anticancer effects of 17-DMAG against stomach cancer, which prompted this study.

As an HSP90 inhibitor, 17-DMAG competes with ATP at its binding site and, thereby, inhibits the intrinsic ATPase activity of HSP90 [12]. Therefore, inhibition of HSP90 by 17-DMAG could ultimately lead to cell apoptosis by abrogating the cellular processes involving client proteins [13]. Besides destabilizing HSP90 client oncoproteins, GA and its derivatives exert cytotoxicity by generating reactive oxygen species (ROS). There are two explanations for this mechanism [14, 15]. First, GA derivatives contain a quinone group, which eventually forms ROS-generating radicals after reacting with flavin-containing reductases and ascorbates. In the body, the one or two electron reduction of quinone to semiquinone or hydroquinone is catalyzed by flavoenzymes using reduced nicotinamide adenine dinucleotide/nicotinamide adenine dinucleotide phosphate (NAD[P]H) as electron sources [16]. The semiquinone/hydroquinone radicals can reduce oxygen gas (O_2_) to superoxide (an ROS), which may stimulate cellular oxidative damage [17–20]. Second, HSP90 inhibition by GA derivatives can induce mitochondrial ROS production by elevating endoplasmic reticulum (ER) stress. In this situation, elevated ER stress originates from the accumulation of unfolded protein in the ER. Increased ER stress can disrupt mitochondrial homeostasis and, thereby, stimulate the production of mitochondrial ROS [21].

Our present investigation focused on the potential anticancer effects and underlying mechanism of 17-DMAG against gastric adenocarcinoma. Therefore, we first investigated the anticancer potentials and mechanisms of 17-DMAG against gastric cancer cells and then attempted to determine which mechanism is prominent between canonical (Hsp90-inhibiting) and non-canonical (ROS-generating) pathways.

## RESULTS

### Expression of HSP90 in human gastric cancer cells and tissues

**Figure 1:**
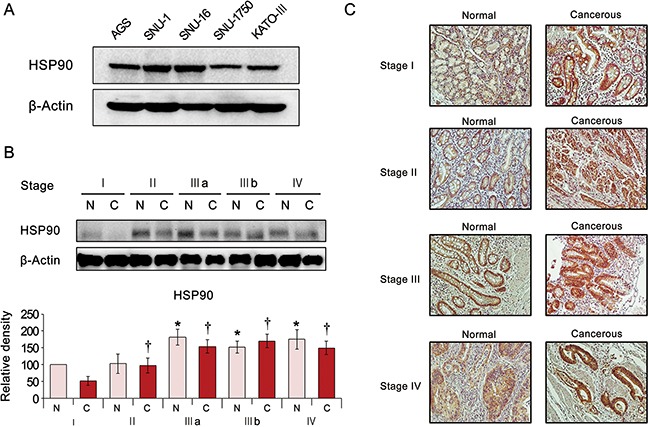
Expression of HSP90 in human gastric cancer cell lines and tissues **(A)** Expression of HSP90 in the various human gastric cancer cell lines. **(B)** **[Top]** Western blot analysis of stomach specimens (N = 12) showing the expression of HSP90 according to tumor stage. Cancerous and adjacent normal stomach tissues from the same patient were termed “cancer” and “normal tissue”, respectively. **[Bottom]** Relative densities of these markers in each group. As tumor stage advanced, the expression of HSP90 increased in both cancer and normal tissues of the same patient. **(C)** HSP90 immunohistochemical staining of stomach specimens according to tumor stage. The expression of HSP90 showed the positive correlation with tumor stage.

We first investigated the expression of HSP90 in various gastric adenocarcinoma cells and discovered it was expressed to a certain degree in the all the cell lines (AGS, SNU-1, SNU-12, SNU-1750, and KATO-III cells) investigated (Figure 1A). To compare the tumor stage-dependent expression of HSP90, we performed comparative western blot analysis of paired surgical specimens (gastric cancer tissues and non-cancerous liver tissues from the same patients) from 12 patients undergoing gastrectomy to treat stomach cancer. Noncancerous and cancerous stomach tissues obtained from patients undergoing gastrectomy were termed “normal” or “cancerous,” respectively. Western blot analysis identified the tumor stage-dependent elevation of the HSP90 expression, which was increased not only in cancerous tissues but also in normal tissues (Figure 1B). Subsequent immunohistochemical staining for HSP90 also revealed the tumor stage-dependent elevation of HSP90 expression (Figure 1C).

### Action mechanism of HSP90 inhibition by 17-DMAG (canonical pathway)

**Figure 2:**
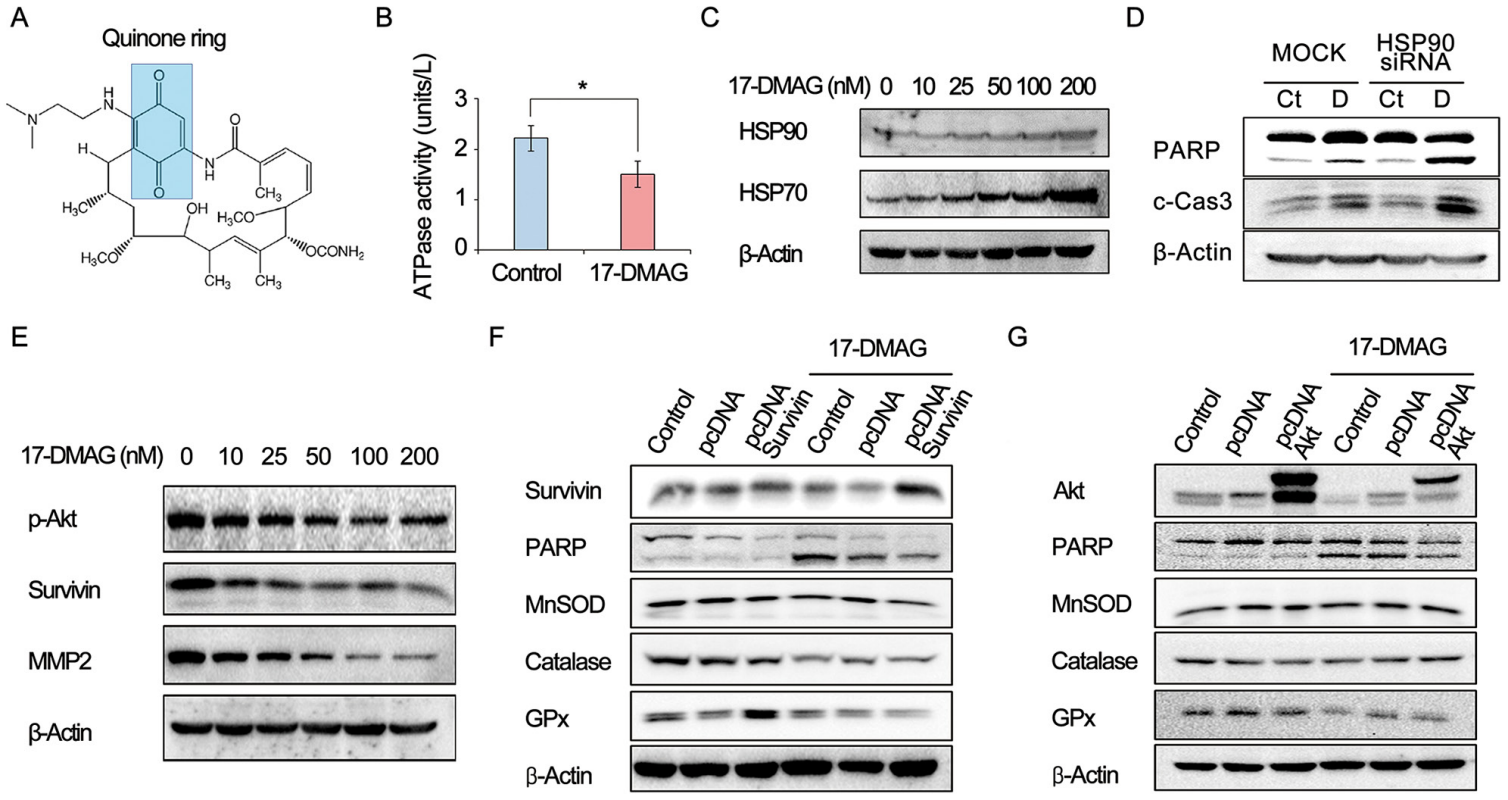
Action mechanism of 17-DMAG as an HSP90 inhibitor (canonical pathway) **(A)** Chemical structure of 17-DMAG which includes a quinone ring. **(B)** HSP90 ATPase activity assay demonstrating the inhibition of ATPase activity of Hsc90 by 17-DMAG. **(C)** Western blot analysis showing dose-dependent elevation of Hsp70 and HSP90 levels in AGS cells by 17-DMAG. **(D)** Western blot analysis to show efficiency of HSP90 siRNA. HSP90 inhibition by HSP90 siRNA promoted proapoptotic effects of 17-DMAG. **(E)** Western blot analysis showing the dose-dependent reduction of the expression of HSP90 client proteins, such as p-Akt, survivin, and MMP2 by 17-DMAG. **(F-G)** Western blot analysis to determine the anticancer effects as an HSP90 inhibitor (canonical pathway). Overexpressing HSP90 client proteins, such as survivin and Akt, did not considerably reduced the apoptosis of AGS cells. In addition, overexpressing the client proteins did not abrogate the potential of 17-DMAG reducing the expression of antioxidant enzymes (catalase, GPx, and MnSOD). Values represent means ± SD of three independent experiments. PARP, polyADP-ribose polymerase; GPx, Glutathione peroxidase; HSP, heat shock protein; MMP2, Matrix metalloproteinase 2; MnSOD, Manganase superoxide dismutase. *P < 0.05.

17-DMAG, an HSP90 inhibitor, is a synthetic analog of the benzoquinone ansamycin antibiotic geldanamycin (Figure 2A). An HSP90 ATPase activity assay indicated that 17-DMAG inhibits the ATPase activity of HSP90 by binding to its ATPase domain (Figure 2B). 17-DMAG dose-dependently increased the HSP70 and HSP90 levels of AGS cells, possibly by a compensatory mechanism activated by their inhibition (Figure 2C). HSP90 inhibition by HSP90 small interfering RNA (siRNA) promoted the proapoptotic effects of 17-DMAG, suggesting that Hsp90 inhibition plays a significant role in the anticancer effects of 17-DMAG (Figure 2D).

As an HSP90 inhibitor, 17-DMAG decreased the expression of HSP90 client proteins such as phosphorylated p-AKT, survivin, and matrix metalloproteinase 2 (MMP2) (Figure 2E). To determine if the anticancer effects involved HSP90 inhibition (canonical pathway), we assessed the proapoptotic effects of 17-DMAG in cells overexpressing representative HSP90 client proteins (survivin and AKT). The overexpression of HSP90 client proteins such as survivin and AKT did not considerably reduce the apoptosis of AGS cells (Figure 2F and 2G). In addition, overexpression of the client proteins did not abrogate the potential of 17-DMAG to reduce the expression of antioxidant enzymes (catalase, glutathione peroxidase [GPx], and manganese superoxide dismutase [MnSOD]). Finally, we investigated cytotoxic effect of proteasome inhibitor MG132 on the proapoptotic effect of 17-DMAG in AGS and SNU-1 cells. Western blot analysis indicated that MG132 promoted proapoptotic effects of 17-DMAG in AGS cells (Supplementary Figure 1). We can think that that disruption of proteasome activity causes accumulation of otherwise degradable proteins within the cell and thus constitutive ER stress causes cell growth arrest, eventually leading to cell death. Taken altogether, the data presented here suggest that 17-DMAG might also act by another independent mechanism (noncanonical pathway) that promotes apoptosis and reduces the expression of antioxidant enzymes. Similar results were obtained in experiments using SNU-1 and KATO- III gastric cancer cells (Supplementary Figure 2).

### 17-DMAG affects proliferation and apoptosis of human gastric cancer cells

**Figure 3:**
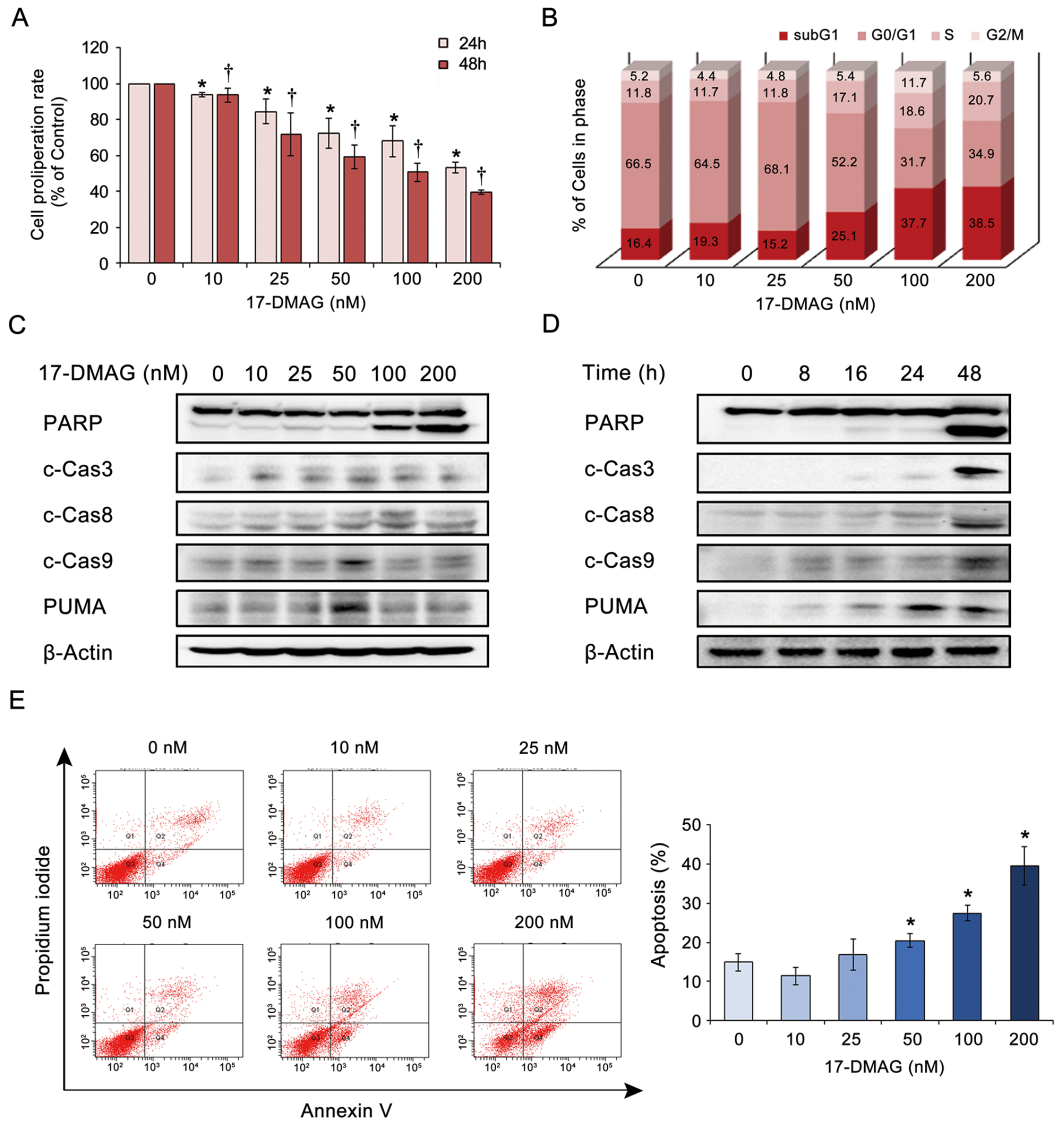
17-DMAG effects on the proliferation and apoptosis of AGS gastric cancer cells **(A)** Proliferation assay of AGS cells treated with graded concentrations of 17-DMAG for 24 h or 48 h. 17-DMAG resulted in significant dose- and time-dependent reduction of AGS cell proliferation (*P* < 0.05). **(B)** 17-DMAG effects on the percentage of apoptotic cells as determined by sub-G1 population (hypodiploid DNA). As the 17-DMAG concentration was raised, the percentage of apoptotic cells increased, up to 38.5%, demonstrating the significant pro-apoptotic effects of 17-DMAG. **(C-D)** 17-DMAG effects on the expression of apoptotic proteins (PARP, c-caspase 3, c-caspase-8, c-caspase-9, and PUMA) in AGS cells. Western blot analyses indicate that 17-DMAG significantly increased the expression of apoptotic proteins in AGS cells in a dose- **(C)** and time- **(D)** dependent manner (*P* < 0.05). **(E)** **[Left]** Quantitative analysis of apoptosis using Annexin V/propidium iodide staining and flow cytometry. Apoptotic cell proportion is expressed as the total percentage of Annexin V-positive cells (early and late apoptotic cells). **[Right]** Relative percentages of apoptotic cells according to varying concentrations of 17-DMAG. The number of Annexin V-positive cells was proportional to the concentration of 17-DMAG (*P* < 0.05). Values represent means ± SD of three independent experiments. c-Cas3, cleaved Caspase 3; c-Cas9, cleaved Caspase 9; PARP, polyADP-ribose polymerase; PUMA, p53 Up-regulated Modulator of Apoptosis. *P < 0.05, † *P* < 0.05.

We investigated the effects of 17-DMAG on the proliferation of AGS gastric cancer cells. The EZ-Cytox proliferation assay revealed that 17-DMAG significantly reduced AGS cell proliferation in a dose- and time-dependent manner (Figure 3A). To estimate the extent of DNA damage induced by 17-DMAG, we quantified the percentage of apoptotic cells (sub-G1 population) induced by varying concentrations of 17-DMAG using flow cytometry (Figure 3B). Increasing the 17-DMAG concentration from 0 to 200 nM increased the percentage of apoptotic cells from 16.4% to 38.5%, respectively, demonstrating the dose-dependent pro-apoptotic effects of 17-DMAG.

We then investigated the effects of 17-DMAG on apoptotic protein levels in AGS cells. Western blot analyses indicated that 17-DMAG significantly increased the expression levels of the apoptotic proteins (polyADP-ribose polymerase [PARP], cleaved-caspase [c-caspase]-3, c-caspase-8, c-caspase-9, and p53 upregulated modulator of apoptosis [PUMA]) in AGS cells in a dose- and time-dependent manner (*P* < 0.05) (Figure 3C and 3D). Finally, the dose-dependent and pro-apoptotic effects of 17-DMAG were also validated by quantitatively measuring apoptosis (the population of Annexin V-positive cells) using Annexin V/propidium iodide (PI) staining and flow cytometry (Figure 3E). Similar results were obtained in experiments using SNU-1 and KATO- III gastric cancer cells (Supplementary Figures 3 and 4).

### Effects of 17-DMAG on ROS levels in human gastric cancer cells

**Figure 4:**
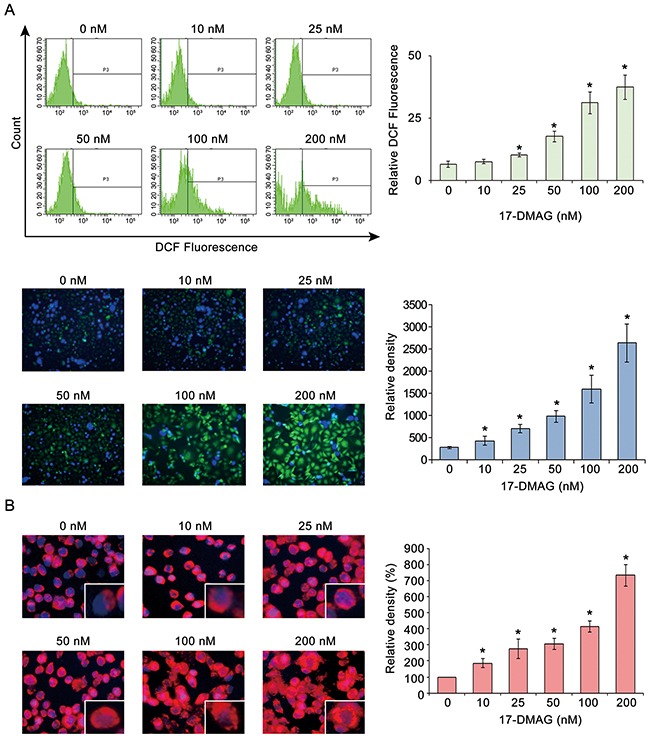
17-DMAG effects on ROS in AGS gastric cancer cells **(A)** Estimation of intracellular ROS levels by flow cytometric analysis of DCF fluorescence intensity **[Top]** and representative fluorescence microphotographs **[Bottom]** of AGS cells with DCF, all of which are proportional to cytosolic ROS levels [6]. All the data indicated that 17-DMAG increased the DCF-fluorescence (ROS levels, green signal) of AGS cells in a dose-dependent manner (*P* < 0.05), showing that 17-DMAG has the potential to elevate ROS levels in gastric cancer cells. **(B)** Quantification of superoxide levels in AGS cells as determined by MitoSOX staining (red signal). As the 17-DMAG concentration was raised, AGS cells more highly demonstrated bright red fluorescence, suggesting that 17-DMAG promotes mitochondrial superoxide synthesis dose-dependently. Values represent means ± SD of three independent experiments. DCF, 2′,7′-dichlorofluorescein. *P < 0.05.

Cancer cells express higher levels ROS levels than other cells do, possibly due to their higher metabolic nature [22]. However, elevation of ROS levels beyond a certain degree could increase the susceptibility of cancer cells to ROS-induced cytotoxicity [22]. Therefore, we investigated the effects of 17-DMAG on the intracellular ROS levels of AGS gastric cancer cells. We estimated intracellular ROS levels using flow cytometric analysis of the fluorescence intensity of dichlorofluorescein (DCF) and microphotographs of AGS cells, which are all proportional to the cytosolic ROS levels [23]. All the data indicated that 17-DMAG increased the DCF fluorescence (ROS levels, green signal) of AGS cells in a dose-dependent manner, showing that 17-DMAG has the potential to elevate ROS levels in gastric cancer cells (Figure 4A). Similar results were obtained in experiments using SNU-1 gastric cancer cells (Supplementary Figure 5).

MitoSOX Red reagent (a red mitochondrial superoxide indicator) is oxidized in the mitochondria by superoxide to subsequently produce red fluorescence [24]. Therefore, we measured mitochondrial ROS levels in AGS cells using MitoSOX. An increase in the 17-DMAG concentration increased the fluorescence intensity of the AGS cells to bright red, suggesting that 17-DMAG dose-dependently promoted mitochondrial superoxide synthesis (Figure 4B).

### Inhibition assay to determine potential ROS-mediated mechanism of action of 17-DMAG

**Figure 5:**
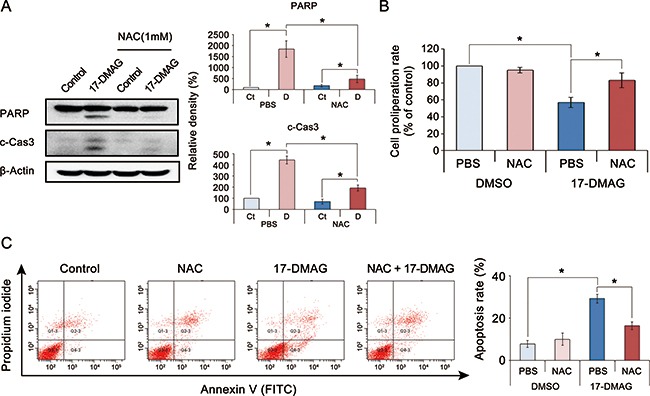
Action mechanism of 17-DMAG with respect to ROS (noncanonical pathway) **(A)** **[Left]** Effects of the ROS inhibitor N-acetyl-L-cysteine (NAC) on the expression of apoptotic proteins of AGS cells treated with 17-DMAG. **[Right]** Relative densities of these markers in each group. Although 17-DMAG increased the expression of apoptotic proteins (PARP and c-Cas3), it was significantly decreased by the addition of NAC (*P* < 0.05). **(B)** Effects of NAC on the proliferation of AGS cells treated with 17-DMAG. The addition of NAC significantly abrogated the proliferation inhibition by 17-DMAG (*P* < 0.05). **(C)** **[Left]** Effects of NAC on the apoptosis of AGS cells treated with 17-DMAG. Apoptotic cells were quantified by counting the total percentage of Annexin V-positive cells. **[Right]** Relative densities of these markers in each group. Although 17-DMAG increased the number of Annexin V-positive cells (early and late apoptotic cells), it was significantly decreased by the addition of NAC (*P* < 0.05). Values represent means ± SD of three independent experiments. Ct, control; DMSO, dimethyl sulfoxide; N, 17-DMAG; NAC, N-acetyl-L-cysteine. *P < 0.05.

To identify the potential ROS-mediated anticancer mechanism of 17-DMAG, we performed an ROS inhibition assay using N-acetyl-L-cysteine (NAC), an ROS inhibitor [25]. We found that NAC abrogated the proapoptotic effects of 17-DMAG in AGS cells (*P* < 0.05) (Figure 5A). The proliferation assay revealed that NAC also abrogated the antiproliferative effects of 17-DMAG in AGS cells (*P* < 0.05) (Figure 5B). To quantify the NAC-induced reduction of apoptosis, we measured apoptosis using Annexin V/PI staining followed by flow cytometry (Figure 5C). Although 17-DMAG increased the numbers of apoptotic cells (population of Annexin V-positive cells), this effect was significantly abrogated by NAC (*P* < 0.05). Similar results were obtained in experiments using SNU-1 gastric cancer cells (Supplementary Figure 6).

NAC inhibits not only oxidative stress but also mTOR pathway [26, 27]. Thus, the inhibition of the antitumor effect of 17-DMAG by NAC can be confounded as the consequence of suppressing the mTOR signaling system. By contrast, another ROS inhibitor, resveratrol has the function of up-regulating phospho–Akt and mTOR levels [28, 29]. We thus performed an ROS inhibition assay using resveratrol (Supplementary Figure 7). The results of western blot analysis indicated that the addition of resveratrol significantly reduced the expression of apoptotic proteins (PARP and c-Cas3) of which expression had been increased by 17-DMAG. Taken altogether, the data presented here suggest that upregulated oxidative stress could be the principal anticancer mechanism of 17-DMAG against gastric cancer cells.

### 17-DMAG affects the expression of antioxidant enzymes and nuclear respiratory factors in gastric cancer cells

**Figure 6:**
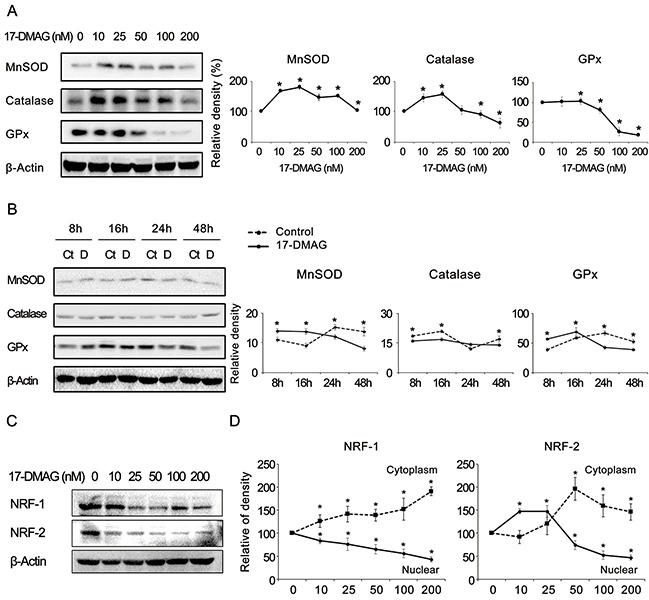
17-DMAG effects on the expression of antioxidant enzymes and NRFs (NRF-1 and NRF-2) in gastric cancer cells (A-B) Western blot analysis to show the effects of 17-DMAG on the expression of antioxidant enzymes 17-DMAG showed the potential of reducing the expression of the antioxidants (MnSOD, catalase, and GPx) both dose-**(A)** and time-**(B)** dependently (*P* < 0.05). **(C)** Western blot analysis showing the 17-DMAG potential of reducing the expression of the NRF-1 and NRF-2 dose-dependently. **(D)** The graph showing the relative densities of the bands of western blot analysis which was performed after separation of cytoplasmic and nucleic fractions of gastric cancer cells. The expression of both NRFs was dose-dependently decreased in the nuclei and increased in the cytoplasm after treatment with 17-DMAG. Values represent means ± SD of three independent experiments. GPx, Glutathione peroxidase; MnSOD, Manganase superoxide dismutase; NRF-1, nuclear respiration factor; NRF-2, nuclear respiration factor-2. *P < 0.05.

To verify the effects of 17-DMAG on the expression of antioxidant enzymes, we examined the expression of representative antioxidants (MnSOD, catalase, and GPx) in AGS gastric cancer cells. The real-time polymerase chain reaction (PCR) experiment revealed that, beyond a certain dose or time, 17-DMAG tended to decrease the mRNA expression of the antioxidants both dose- and time-dependently (Supplementary Figure 8). Western blot analysis also confirmed that 17-DMAG has the potential to reduce the expression of the antioxidants both dose- and time-dependently (Figure 6A and 6B). Similar results were obtained in experiments using SNU-1 and KATO- III gastric cancer cells (Supplementary Figures 9 and 10). In addition, 17-DMAG induced the significantly higher GSSH/GSH ratio in both gastric cancer cell lines, demonstrating that 17-DMAG significantly decreased the intracellular glutathione levels (Supplementary Figure 11).

Subsequently, we investigated the effects of 17-DMAG on the expression of NRF-1 and NRF-2 because they are known representative redox-sensitive transcription factors that regulate the expression of diverse antioxidant genes [30–33]. The western blot analysis revealed that 17-DMAG showed the potential to reduce the expression of NRFs dose-dependently in various gastric cancer cell lines, including AGS cells (Figure 6C) as well as SNU-1 and KATO-III cells (Supplementary Figure 12). Furthermore, to determine whether 17-DMAG affects the translocation of NRFs from the nucleus to the cytoplasm, we performed western blot analyses of cytoplasmic and nuclear fractions of gastric cancer cells, separately (Supplementary Figure 13). Figure 6D shows the relative densities of the western blot bands, which indicates that the expression of both NRFs was dose-dependently decreased in the nuclei and increased in the cytoplasm after treatment with 17-DMAG. Collectively, our results suggest that 17-DMAG not only reduced the NRF expression but also prevented NRFs from migrating from the cytoplasm to the nucleus. Similar results were obtained in experiments using SNU-1 gastric cancer cells (Supplementary Figure 14).

### Effects of 17-DMAG on antioxidant enzyme expression in specimens from patients with gastric adenocarcinoma

**Figure 7:**
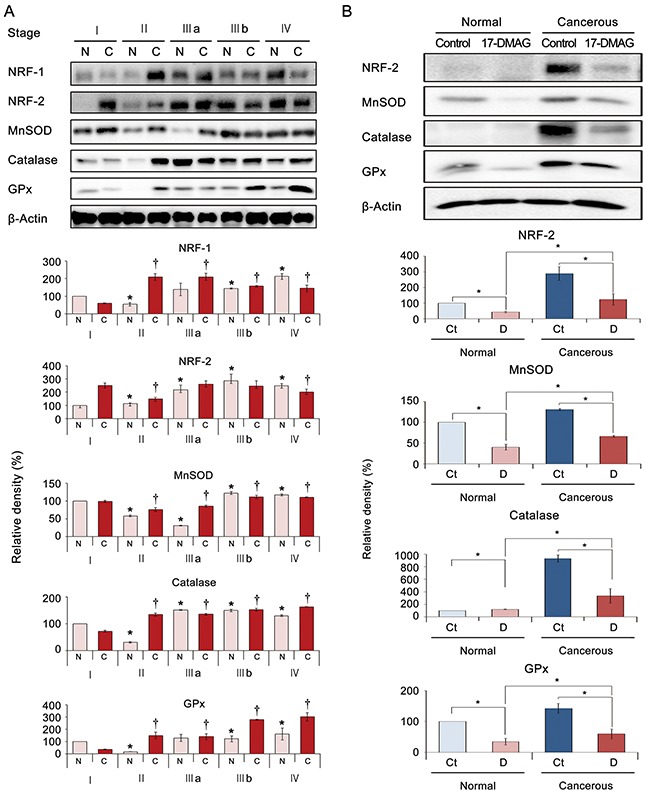
17-DMAG effects relating to the expression of antioxidant enzymes in specimens of patients with gastric adenocarcinoma Cancerous and adjacent noncancerous stomach tissues obtained from patients undergoing gastrectomy (N = 12) were termed “cancer” and “normal tissues”, respectively. **(A)** **[Top]** Western blot analyses of stomach specimens showing the expression of antioxidant-related markers, including NRFs and antioxidant proteins (MnSOD, catalase, and GPx) according to tumor stage. **[Bottom]** Relative densities of these markers in each group. As tumor stage advanced, the expression of these markers increased in both normal and cancer tissues of the same patient. **(B)** **[Top]** 17-DMAG effects on the expression of antioxidant-related markers in an *ex vivo* model of gastric adenocarcinoma. The *ex vivo* model was established by culturing minced gastric cancer tissues (N = 6) with or without 100 nM 17-DMAG for 24 h. **[Bottom]** Relative densities of these markers in each group. Western blot analysis demonstrated that 17-DMAG significantly decreased the expression of antioxidant-related markers (NRF-2, MnSOD, catalase, and GPx) in both normal and cancer tissues (all *P*'s < 0.05). Values represent means ± SD of three independent experiments. Ct, Control; D, 17-DMAG; GPx, Glutathione peroxidase; MnSOD, Manganase superoxide dismutase; NRF-1, nuclear respiration factor; NRF-2, nuclear respiration factor-2. *P < 0.05, † *P* < 0.05.

To determine the clinical correlation of the effects of 17-DMAG, the expression patterns of the NRFs and antioxidant enzymes were investigated in patient surgical specimens (N = 12) depending on the tumor stage. Western blot analysis identified the tumor stage-dependent elevation of NRF expression and antioxidant enzyme (MnSOD, catalase, and GPx) activity. Interestingly, the expression of these markers was increased in both cancer tissues as well as the adjacent normal stomach tissues (Figure 7A).

Next, we investigated the effects of 17-DMAG on the expression of NRF-1 and antioxidant enzymes in *ex vivo* cultures of gastric tissues obtained from patients who underwent gastrectomies (paired normal stomach and gastric adenocarcinoma tissues from each patient, N = 10). The *ex vivo* model was established by culturing minced gastric cancer tissues with or without 100 nM 17-DMAG for 24 h immediately following gastrectomy. Western blot analysis demonstrated that 17-DMAG significantly decreased the expression of NRF-2 and the antioxidant enzymes in both noncancerous and cancerous gastric tissues (*P* < 0.05) (Figure 7B).

### Effects of 17-DMAG on AGS cell xenograft growth in nude mice

**Figure 8:**
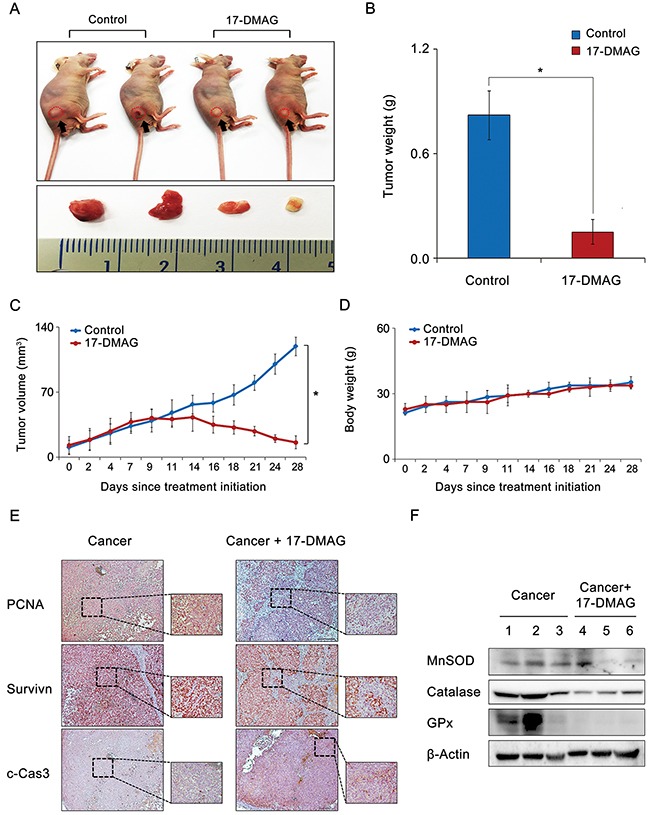
Effects of 17-DMAG on the growth of AGS cells xenografted in nude mice After 17-DMAG (10 mg/kg/day) was administered intraperitoneally 3 times a week, mice were euthanized, and their tumors were collected. **(A)** Morphological images of mice with xenografted AGS cells and tumors in each group. Images of tumors before and after necropsy show that tumor shrinkage was more prominent in mice treated with 17-DMAG than in control mice (*P* < 0.05). **(B)** Final tumor weight at 4 weeks. A considerably greater reduction in tumor weight was observed in mice treated with 17-DMAG than in control mice (*P* < 0.05). **(C)** Tumor volumes were calculated according to the formula: volume = length × width^2^ × 0.5236. Note that a significant reduction in tumor volumes was detected in mice treated with 17-DMAG compared with control mice (*P* < 0.05). **(D)** Body weight changes during the experiment. The average body weight of mice in each group did not vary significantly over the course of the experiment. **(E)** Immunohistochemical stains demonstrating that 17-DMAG treated group exhibited the negative rates of PCNA and survivin expression and the positive rates of c-Caspase 9 expression. **(F)** Western blot analysis showing that 17-DMAG treated group exhibited significantly lesser expression of antioxidant enzymes than did the control group. c-Cas3: cleaved-caspase 3, GPx, Glutathione peroxidase; MnSOD, Manganase superoxide dismutase; PCNA, Proliferating cell nuclear antigen. *P < 0.05.

We examined the potential 17-DMAG-induced inhibition of the growth of AGS cell xenografts in nude mice. After a 4-week intraperitoneal administration of 17-DMAG (10 mg/kg in 100 μL normal saline three times a week), the mice were euthanized, and their tumors were collected. Images of the tumors before and after necropsy showed that the shrinkage was more prominent in mice treated with 17-DMAG than it was in the control mice (Figure 8A). A more considerable reduction in tumor weight was observed in mice treated with 17-DMAG than in the control mice (*P* < 0.05) (Figure 8B). Mice treated with 17-DMAG exhibited a statistically significant reduction in tumor volume compared with that in the control mice (*P* < 0.05) (Figure 8C). However, the average body weight of mice in each treatment group did not vary significantly throughout the experiment (Figure 8D). Collectively, the data presented here indicate that 17-DMAG had the potential to reduce the growth of xenografted AGS cells in nude mice without causing any observable side effects.

Finally, we performed histological and molecular investigations on the tumor cell mass obtained from the xenograft model. The immunohistochemical staining revealed that the 17-DMAG-treated group exhibited a decrease in the proliferating cell nuclear antigen (PCNA) and survivin expression, and an increase in c-caspase 3 expression (Figure 8E). Subsequently, the western blot analysis showed that the 17-DMAG-treated group exhibited significantly lower expression levels of the antioxidant enzymes than the control group did (*P* < 0.05) (Figure 8F).

## DISCUSSION

**Figure 9:**
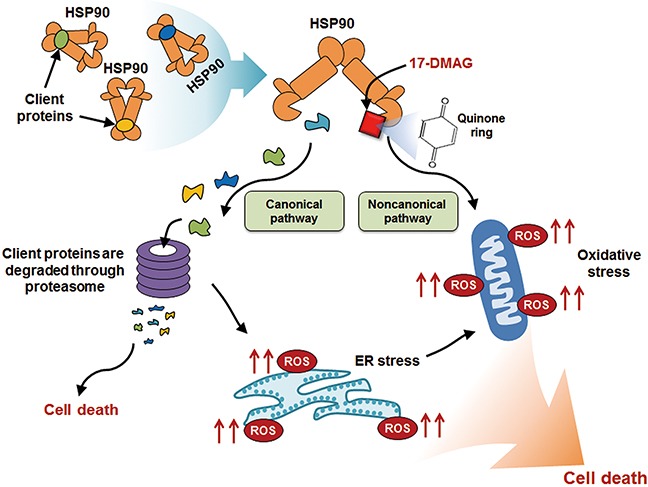
Possible anticancer action mechanism of 17-DMAG against gastric cancer cells The anticancer mechanisms of 17-DMAG includes canonical and noncanonical pathways. In canonical pathway, 17-DMAG inhibits ATPase activity of HSP90 which stabilizes numerous oncoproteins. The client proteins which are not stabilized by HSP00 are degraded by the proteasome. In noncanonical pathway, 17-DMAG exerts its anticancer effects by altering oxidant-antioxidant balance which includes elevating ROS levels while decreasing the levels of antioxidant enzymes. The elevated ROS generation by 17-DMAG can be attributed to its quinone ring-bearing structure, which eventually forms ROS-generating radicals after reacting with flavin-containing reductases and ascorbates. In addition, HSP90 inhibition by 17-DMAG could induce ROS production, possibly as a consequence of elevated ER stress. Abbreviations: ER, Endoplasmic reticulum; HSP90, Heat shock protein 90; ROS, Reactive oxygen species.

Our investigation focused on determining the anticancer effects of 17-DMAG against gastric cancer and its underlying mechanisms of action. We demonstrated that 17-DMAG exhibited significant antiproliferative and proapoptotic effects against gastric cancer in the various *in vitro, ex vivo*, and *in vivo* anticancer models. The anticancer mechanisms of 17-DMAG included canonical and noncanonical pathways, which were evidenced by inhibition of HSP90 and ROS generation, respectively. Analysis of the canonical pathway demonstrated that 17-DMAG significantly inhibited the ATPase activity of HSP90 in gastric cancer cells. Analysis of the noncanonical pathway revealed that 17-DMAG increased ROS levels while decreasing the expression of antioxidant enzymes in gastric cancer cells. Moreover, 17-DMAG significantly decreased the expression of NRF-1 and NRF-2, which is essential for the transcription of antioxidant enzymes. Of the two mechanisms, we attempted to find the dominant anti-gastric cancer mechanism of 17-DMAG. Abrogating the canonical pathway by overexpressing representative HSP90 client proteins did not significantly reduce the proapoptotic effects of 17-DMAG. However, abrogating the noncanonical pathway using an ROS inhibitor (NAC) significantly decreased the anticancer potential of 17-DMAG. Taken altogether, our results strongly suggest that 17-DMAG exerts its anticancer effects against gastric cancer cells principally through the noncanonical pathway, which was manifested by both up- and down-regulation of ROS and antioxidant enzymes, respectively. Figure 9 shows the possible anticancer mechanism of 17-DMAG against gastric cancer cells.

We found that HSP90 expression was positively correlated with the tumor stage of gastric cancer. In addition, as the tumor stage advanced, the expression of HSP90 was increased not only in cancer tissues but also in the surrounding normal gastric tissue of the patients. Gastric carcinogenesis is a multistep process [34], and precancerous conditions usually precede the development of gastric adenocarcinoma [35]. For instance, well-differentiated adenocarcinoma is established through the following three subpathways: 1) an intestinal metaplasia-adenoma-carcinoma sequence, 2) an intestinal metaplasia-carcinoma sequence, and 3) de novo carcinogenesis [36]. The pericancerous tissue is at risk for transitioning to intestinal metaplasia and ultimately to carcinoma. Our study suggests that the pericancerous tissue exhibited a higher HSP90 expression level as it is undergoing the multistep carcinogenesis process. Thus, as an HSP90 inhibitor, 17-DMAG is expected to inhibit not only the progression of gastric cancer but also the transition from precancerous lesions to carcinomas.

This study shows that the anticancer activity of 17-DMAG considerably depended on the promotion of ROS generation in gastric cancer cells. 17-DMAG is a bioactive analog of GA, which has a quinone group in common with 17-DMAG and its other analogs. Quinones induce biological toxicity based on their arylation of biological nucleophiles, induction of redox-cycling reactions to generate ROS, or both [37]. In biological systems, NAD(P)H-dependent flavoenzymes catalyze the reduction of quinone either into semiquinone or hydroquinone radicals, which can generate ROS [38, 39]. Molecules containing a quinone group have redox-active properties [40, 41]. For instance, streptonigrin, an ansamycin antibiotic similar to GA, can degrade the deoxyribose of DNA via superoxide and hydroxyl-like radicals generated by semiquinones [42]. In addition, the hepatotoxicity of GA and its derivatives is mediated by the redox-cycling quinone structure of GA [43].

The results of our study are also in agreement with those of a study showing that the HSP90 inhibitor, NVP-AUY922, induced cell apoptosis by inducing mitochondrial damage, which was prompted by ER stress [21]. Taiyab et al. also showed that HSP90 inhibition induces apoptosis through ER stress in addition to inhibiting cell proliferation caused by cell cycle arrest. Further, they identified the mechanism by which ER stress disrupts mitochondrial homeostasis and thus induces apoptosis of rat histiocytoma cells. When the cellular insult is not severe, it initiates the adaptive pathway, which appropriately defends against injuries including initiating measures that restore ER homeostasis. However, when the cellular insult rises to a certain degree, it eventually activates the proapoptotic pathway [44, 45]. Most cancer cells are well known to exhibit chronically elevated ER stress levels [46]. Therefore, the induction of cancer cell apoptosis using medications that specifically trigger ER stress could be an attractive anticancer strategy [47]. Inhibiting HSP90 leads to increased unfolded protein accumulation and, subsequently, increased ER stress. ER stress subsequently induces mitochondrial ROS production by inducing Ca^2+^ release and depolarizing the inner mitochondrial membrane [21, 48]. Therefore, increased ER stress can ultimately induce cell death by promoting ROS production.

Our experiments also showed that 17-DMAG dose-dependently decreased the expression of NRFs. Moreover, 17-DMAG prevented NRFs from migrating from the cytoplasm to the nucleus dose-dependently. NRF-1 and NRF-2 are master regulators of oxidative stress-induced gene expression [49–51]. The transcriptional regulatory regions of antioxidant enzyme genes have the Maf recognition element-related sequence that is referred to as the antioxidant or electrophile response element (ARE/EpRE) [52, 53]. The expression of ARE/EpRE-bearing genes was severely impaired in fibroblasts deficient in NRF-1 and NRF-2, resulting in marked sensitivity to oxidative stress. This suggests that NRFs are the transcription factors that regulate the expression of antioxidant enzymes through ARE/EpRE. Under normal homeostatic redox conditions, NRF-2 is sequestered by Kelch-like ECH-associated protein 1 (Keap1) in the cytoplasm where it negatively regulates NRF-2 through ubiquitin-mediated proteasomal degradation [54]. However, following ROS stimulation, Keap1 releases NRF-2 to enable its nuclear localization and transactivation via the ARE/EpRE sequences. Together, these studies indicate that NRF-1 and NRF-2 have overlapping functions and mediate the expression of ARE/EpRE-bearing genes during oxidative stress responses. Our results suggest that 17-DMAG strongly inhibited the expression of antioxidant enzymes both by suppressing the expression of NRFs and by preventing NRFs from migrating from the cytoplasm to the nucleus. Consequently, reduced expression of antioxidant enzymes abrogates the appropriate disposal of increased ROS.

In conclusion, this study provides evidence that 17-DMAG exerts its anticancer effects against gastric cancer principally through the noncanonical pathway, which is manifested by up- and downregulation of ROS and antioxidant enzymes, respectively. We also showed that 17-DMAG strongly inhibits the expression of antioxidant enzymes by suppressing the expression of both NRF-1 and NRF-2, and that the consequent reduction in expression of antioxidant enzymes results in a failure to inactivate ROS. The elevated ROS generation by 17-DMAG can be attributed to its quinone ring-bearing structure, which eventually forms ROS-generating radicals after reacting with flavin-containing reductases and ascorbates. In addition, HSP90 inhibition by 17-DMAG could induce mitochondrial ROS production, possibly as a consequence of elevated ER stress. Therefore, this study lays the foundation for a novel, oxidant/antioxidant-based therapeutic approach to the treatment of gastric cancer. Clinical studies are required to identify patient populations that would significantly benefit from 17-DMAG-based therapy.

## MATERIALS AND METHODS

### Chemicals and reagents

17-DMAG was obtained from Selleckem (Farmingdale, NY, USA); 2′,7′-dichlorodihydrofluorescein diacetate (DCF-DA) was purchased from Thermo Fisher Scientific (San Jose, CA, USA); N-acetyl-L-cysteine (NAC) was obtained from Sigma Aldrich (St Louis, MO, USA); SYBR Green was obtained from Promega (Madison, WI, USA); and, Hsp90 siRNA was obtained from Santa Cruz Biotechnology (Dallas, TX, USA).

### Cell culture

Human gastric adenocarcinoma cell lines (AGS, SNU-1, KATO-III, SNU-16, and SNU-1750) were obtained from KCLB (Korean cell line bank). Cells were maintained in RPMI 1640 medium (Thermo Fisher Scientific). The medium was supplemented with 10% heat inactivated fetal bovine serum (FBS; Thermo Fisher Scientific) and 1% penicillin-streptomycin antibiotics (Thermo Fisher Scientific) at 37°C in a humidified atmosphere with 5% CO_2_ in incubator.

### Colorimetric determination of Hsp90 ATPase activity

Hsp90 ATPase activity was monitored by phosphate hydrolysis using ATPase activity assay kit (Sigma Aldrich) according to the manufacturer's instruction. Briefly, Hsp90 inhibitor treated samples incubated for 0.5 hours at 25°C with 4 mM ATP. After incubation, the absorbance of colorimetric product was measured at 620 nm using the microplate reader (Bio-Rad, Hercules, CA, USA).

### Cell proliferation assay

Cell proliferation was measured by EZ-Cytox Cell Proliferation Assay kit (Itsbio, Seoul, Republic of Korea) according to the manufacturer's instruction. Briefly, AGS, SNU-1, and KATO- III cells were plated in 96-well plates and cultured overnight (1 × 10^4^ cells per well). The cells were treated with 17-DMAG of different concentrations (10,25,50,100 and 200 nM) for 24 h and 48 h, respectively. Then, the reagent from EZ-Cytox Cell Proliferation Assay kit was applied to each well. Absorbance was measured at 450 nm using a microplate reader (model 680; Bio-Rad).

### GSSG/GSH assay

The levels of reduced glutathione (GSH) and oxidized glutathione (GSSG) were measured using an OxiSelect Glutathione Assay kit (Cell Biolabs), according to the manufacturer's instruction. Briefly, gastric cancer cells treated with 17-DMAG (100 nM) for 48 h, harvest cell was homogenized in 5% metaphosphoric acid, and the supernatant was collected for assay. After incubation, the absorbance measured at 405 nm at 1 min interval for 10 min using the microplate reader (Bio-Rad).

### Cell cycle analysis

After treatment with 17-DMAG, gastric cancer cells were harvested in cold phosphate-buffered saline (PBS), fixed in 70% ethanol, stored overnight at 4°C, and resuspended in 50 μg/ml propidium iodide (PI) staining reagent. Cells were incubated for 30 min in the dark at 25°C, and then cell cycle analysis was conducted using a BD FACSCANTO II cytometer (BD Biosciences, San Jose, CA, USA).

### Western blotting analysis

Gastric cancer cells (AGS, SNU-1, and KATO-III cells) and patient-derived gastric tissues were lysed using the EzRIPA Lysis kit (ATTO Corporation; Tokyo, Japan), and quantified with Bradford reagent (Bio-Rad). Proteins were visualized by western analysis using primary antibodies (1:1,000 dilution) at 4°C overnight followed by HRP-conjugated secondary antibodies (1:2,000 dilution) for 1 h at 25°C. The details of antibodies obtained from Cell Signaling Technology (Danvers, MA, USA) are as follows: primary antibodies against cleaved Poly ADP-Ribose Polymerase (PARP), cleaved caspase-3 (c-caspase-3), cleaved caspase-8 (c-caspase-3), cleaved caspase-9 (c-caspase-3), p53 Up-regulated Modulator of Apoptosis (PUMA), nuclear respiratory factor-1 (NRF-1), NRF-2, manganese superoxide dismutase (MnSOD), catalase, glutathione peroxidase (GPx), lamin B1, and β-actin, as well as Horseradish peroxidase (HRP)-conjugated secondary antibody. Specific immune complexes were detected using Western Blotting Plus Chemiluminescence Reagent (Millipore, Bedford, MA, USA).

### Quantification of cell ros and apoptosis by flow cytometry

To detect ROS and apoptosis, cells were stained with either DCF-DA or Annexin V/PI using the FITC Annexin V apoptosis detection kit (BD Biosciences), respectively. After incubation for 15 min in the dark at 25°C, the cells were analyzed using a BD FACSCANTO II cytometer (BD Biosciences).

### Detection of cell mitochondrial superoxide by MitoSOX staining

To detect mitochondrial ROS, cells were stained with MitoSOX (Molecular Probes, Waltham, MA, USA). After incubation for 10 min in the dark at 37°C, the cells were analyzed using a laser-scanning microscope (Eclipse TE300; Nikon, Tokyo, Japan).

### Preparation of nuclear and cytosol fractionation

Nuclear and cytosolic fractions of AGS cells were obtained using a commercially available nuclear/cytosol fraction kit (Biovision, CA, USA). Briefly, AGS cells were homogenized in cytosolic extraction reagent containing protease inhibitor cocktail. After a series of washing steps, cytosolic extract was obtained by centrifugation at 16,000 ×g for 10 min. Nuclear proteins were extracted in nuclear extraction reagent containing protease inhibitor cocktail. Nuclear extracts were obtained by centrifugation at 16,000 ×g for 10 min.

### Immunohistochemistry

Paraffin-embedded tissue sections were deparaffinized in xylene and rehydrated in a graded series of alcohol. Antigens were retrieved with 0.01 M citrate buffer (pH 6.0) by heating the samples in a microwave oven for 10 min. The tissue sections were then placed in 3% hydrogen peroxide for 5 min to inactivate endogenous peroxidase, and blocked for 30 min with normal horse serum (VECTASTAIN Elite ABC kit; Vector Laboratories, Burlingame, CA, USA). The primary antibody used for this study was anti-Hsp90 antibody (Cell Signaling). Pre-diluted primary antibodies were applied overnight at 4°C. The slides were then treated with biotinylated secondary antibody for 30 min at room temperature, followed by immPACT NovaRED Peroxidase substrate (Vector Laboratories) for 5 min at room temperature.

### Quantitative real-time PCR

Total RNA was extracted from cells using TRIzol reagent (Invitrogen) according to the manufacturer`s instructions. Reverse transcription was performed with 1 μg of RNA, random primers, and M-MLV Reverse transcriptase (Promega, MI, USA). The primers used for SYBR Green reverse transcription-qPCR (RT-qPCR) were as follows: MnSOD forward *5′-*GCATCTGTTGGTGTCCAAGG*-3′* and reverse *5′ -*CTGTTGTTCCTTGCAGTGG*-3′*; Catalase forward *5′-*GAAGGATCCGGACATGGTCT *-3′* and reverse *5′-* GATGTCCATCTG GAATCCCC *-3′*; GPx, forward *5′-* TCGAGAAGTGCGAGGTGAAC *-3′* and reverse *5′-*AGCT TGGGGTCGGTCATAAG *-3′*; and GAPDH, forward *5′-* GCACCGTCAAGGCTGAGAAC-3′ and reverse 5′-TGGTGAAGACGCCAGTGGA-3. RT-qPCR was performed using an Applied Biosystems® 7500 Fast Real-Time PCR System(Life Technologies, Carlsbad, CA, USA) equipped with a 96-well optical reaction plate. After normalization to the GAPDH gene, expression levels for each target gene were calculated using the comparative threshold cycle method. Data were presented as the mean ± standard deviation (SD) from three independent experiments.

### Obtaining patient-derived tissues and *ex vivo* model of HCC tissues

Human gastric cancer tissue specimens (paired normal and cancer tissues from each patient, N = 12) were obtained after surgical resections performed at our institution. The ethics committee at our institution approved the use of the tissue specimens for the research (IRB No: DC17SISI.0001).

For the *ex vivo* culture modeling of gastric cancer tissues, another patient samples of gastric cancer tissue specimens (N = 10) were obtained immediately following gastrectomy. They were washed thrice, dissected in cold PBS (Invitrogen) containing 2× penicillin/streptomycin mixture (Thermo Fisher Scientific), and cultured in 24-well culture plates with serum-free DMEM/F12 containing 2× penicillin/streptomycin and treated with 17-DMAG, for 24 h to establish an *ex vivo* culture model for gastric cancer tissues. The plates were then placed in a humidified incubator and maintained in an atmosphere of 5% CO_2_ at 37°C.

### *In vivo* xenograft model

BALB/c nude mice (6 weeks) were used for comparative modeling of subcutaneous tumor growth. AGS cells (10^7^ cells) and Matrigel (BD Biosciences) were subcutaneously injected into each mouse. The mice were weighed twice per week. Fourteen days after tumor cell injection, all mice had measurable tumors. Mice were then randomly grouped (n = 10 per group), and treated intraperitoneally with either normal saline (control) or 17-DMAG (10 mg/kg in 100 μL normal saline, 3 times a week), for 25 days. Tumor sizes were measured twice weekly with calipers, and tumor volume was calculated using the formula: volume = length × width^2^ × 0.5236 [55]. After completion of treatment, all mice were euthanized.

### Statistical analysis

All data were analyzed using SPSS 11.0 software (SPSS Inc.; Chicago, IL, USA) and were presented as the mean ± standard deviation (SD). Statistical comparisons between the mean values of two groups were performed using the Mann-Whitney U-test, whereas for comparison of three or more groups, the Kruskal-Wallis test was used. Probability (*P*) values of < 0.05 were considered statistically significant.

## SUPPLEMENTARY MATERIALS FIGURES



## References

[1] International Agency for Research on Cancer (2014). Globocan 2012: Estimated cancer incidence, mortality and prevalence worldwide in 2012. International Agency for Research on Cancer.

[2] Chrom P, Stec R, Szczylik C (2015). Second-line treatment of advanced gastric cancer: current options and future perspectives. Anticancer Res.

[3] Shirota T, Ojima H, Hiraoka N, Shimada K, Rokutan H, Arai Y, Kanai Y, Miyagawa S, Shibata T (2015). Heat shock protein 90 is a potential therapeutic target in cholangiocarcinoma. Mol Cancer Ther.

[4] Chen MH, Chiang KC, Cheng CT, Huang SC, Chen YY, Chen TW, Yeh TS, Jan YY, Wang HM, Weng JJ, Chang PM, Liu CY, Li CP (2014). Antitumor activity of the combination of an HSP90 inhibitor and a PI3K/mTOR dual inhibitor against cholangiocarcinoma. Oncotarget.

[5] Pick E, Kluger Y, Giltnane JM, Moeder C, Camp RL, Rimm DL, Kluger HM (2007). High HSP90 expression is associated with decreased survival in breast cancer. Cancer Res.

[6] Song CH, Park SY, Eom KY, Kim JH, Kim SW, Kim JS, Kim IA (2010). Potential prognostic value of heat-shock protein 90 in the presence of phosphatidylinositol-3-kinase overexpression or loss of PTEN, in invasive breast cancers. Breast Cancer Res.

[7] Neckers L, Neckers K (2002). Heat-shock protein 90 inhibitors as novel cancer chemotherapeutic agents. Expert Opin Emerg Drugs.

[8] Workman P, Burrows F, Neckers L, Rosen N (2007). Drugging the cancer chaperone HSP90: combinatorial therapeutic exploitation of oncogene addiction and tumor stress. Ann N Y Acad Sci.

[9] Jhaveri K, Miller K, Rosen L, Schneider B, Chap L, Hannah A, Zhong Z, Ma W, Hudis C, Modi S (2012). A phase I dose-escalation trial of trastuzumab and alvespimycin hydrochloride (KOS-1022; 17 DMAG) in the treatment of advanced solid tumors. Clin Cancer Res.

[10] Eiseman JL, Lan J, Lagattuta TF, Hamburger DR, Joseph E, Covey JM, Egorin MJ (2005). Pharmacokinetics and pharmacodynamics of 17-demethoxy 17-[[(2-dimethylamino)ethyl]amino]geldanamycin (17DMAG, NSC 707545) in C. B-17 SCID mice bearing MDA-MB-231 human breast cancer xenografts. Cancer Chemother Pharmacol.

[11] Pacey S, Wilson RH, Walton M, Eatock MM, Hardcastle A, Zetterlund A, Arkenau HT, Moreno-Farre J, Banerji U, Roels B, Peachey H, Aherne W, de Bono JS (2011). A phase I study of the heat shock protein 90 inhibitor alvespimycin (17-DMAG) given intravenously to patients with advanced solid tumors. Clin Cancer Res.

[12] Stebbins CE, Russo AA, Schneider C, Rosen N, Hartl FU, Pavletich NP (1997). Crystal structure of an Hsp90-geldanamycin complex: targeting of a protein chaperone by an antitumor agent. Cell.

[13] Burrows F, Zhang H, Kamal A (2004). Hsp90 activation and cell cycle regulation. Cell Cycle.

[14] Dikalov S, Landmesser U, Harrison DG (2002). Geldanamycin leads to superoxide formation by enzymatic and non-enzymatic redox cycling. Implications for studies of Hsp90 and endothelial cell nitric-oxide synthase. J Biol Chem.

[15] Sreedhar AS, Mihaly K, Pato B, Schnaider T, Stetak A, Kis-Petik K, Fidy J, Simonics T, Maraz A, Csermely P (2003). Hsp90 inhibition accelerates cell lysis. Anti-Hsp90 ribozyme reveals a complex mechanism of Hsp90 inhibitors involving both superoxide- and Hsp90-dependent events. J Biol Chem.

[16] Deller S, Macheroux P, Sollner S (2008). Flavin-dependent quinone reductases. Cell Mol Life Sci.

[17] Beckman JS, Beckman TW, Chen J, Marshall PA, Freeman BA (1990). Apparent hydroxyl radical production by peroxynitrite: implications for endothelial injury from nitric oxide and superoxide. Proc Natl Acad Sci U S A.

[18] Dedon PC, Tannenbaum SR (2004). Reactive nitrogen species in the chemical biology of inflammation. Arch Biochem Biophys.

[19] Goldstein S, Lind J, Merenyi G (2005). Chemistry of peroxynitrites as compared to peroxynitrates. Chem Rev.

[20] Radi R (2004). Nitric oxide, oxidants, and protein tyrosine nitration. Proc Natl Acad Sci U S A.

[21] Taiyab A, Sreedhar AS, Rao Ch M (2009). Hsp90 inhibitors, GA and 17AAG, lead to ER stress-induced apoptosis in rat histiocytoma. Biochem Pharmacol.

[22] Yang Y, Karakhanova S, Werner J, Bazhin AV (2013). Reactive oxygen species in cancer biology and anticancer therapy. Curr Med Chem.

[23] Lin YL, Wu CH, Luo MH, Huang YJ, Wang CN, Shiao MS, Huang YT (2006). *In vitro* protective effects of salvianolic acid B on primary hepatocytes and hepatic stellate cells. J Ethnopharmacol.

[24] Zhang L, Seitz LC, Abramczyk AM, Chan C (2010). Synergistic effect of cAMP and palmitate in promoting altered mitochondrial function and cell death in HepG2 cells. Exp Cell Res.

[25] Curtin JF, Donovan M, Cotter TG (2002). Regulation and measurement of oxidative stress in apoptosis. J Immunol Methods.

[26] Chen S, Ren Q, Zhang J, Ye Y, Zhang Z, Xu Y, Guo M, Ji H, Xu C, Gu C, Gao W, Huang S, Chen L (2014). N-acetyl-L-cysteine protects against cadmium-induced neuronal apoptosis by inhibiting ROS-dependent activation of Akt/mTOR pathway in mouse brain. Neuropathol Appl Neurobiol.

[27] Leontieva OV, Blagosklonny MV (2011). Yeast-like chronological senescence in mammalian cells: phenomenon, mechanism and pharmacological suppression. Aging (Albany NY).

[28] Liu S, Li T, Liu H, Wang X, Bo S, Xie Y, Bai X, Wu L, Wang Z, Liu D (2016). Resveratrol exerts antidepressant properties in the chronic unpredictable mild stress model through the regulation of oxidative stress and mTOR pathway in the rat hippocampus and prefrontal cortex. Behav Brain Res.

[29] Saldanha JF, Leal Vde O, Stenvinkel P, Carraro-Eduardo JC, Mafra D (2013). Resveratrol: why is it a promising therapy for chronic kidney disease patients?. Oxid Med Cell Longev.

[30] Kim SK, Yang JW, Kim MR, Roh SH, Kim HG, Lee KY, Jeong HG, Kang KW (2008). Increased expression of Nrf2/ARE-dependent anti-oxidant proteins in tamoxifen-resistant breast cancer cells. Free Radic Biol Med.

[31] Osburn WO, Kensler TW (2008). Nrf2 signaling: an adaptive response pathway for protection against environmental toxic insults. Mutat Res.

[32] Venugopal R, Jaiswal AK (1998). Nrf2 and Nrf1 in association with Jun proteins regulate antioxidant response element-mediated expression and coordinated induction of genes encoding detoxifying enzymes. Oncogene.

[33] Wang W, Kwok AM, Chan JY (2007). The p65 isoform of Nrf1 is a dominant negative inhibitor of ARE-mediated transcription. J Biol Chem.

[34] Correa P (1988). A human model of gastric carcinogenesis. Cancer Res.

[35] Yakirevich E, Resnick MB (2013). Pathology of gastric cancer and its precursor lesions. Gastroenterol Clin North Am.

[36] Tahara E (2004). Genetic pathways of two types of gastric cancer. IARC Sci Publ.

[37] Chang CH, Drechsel DA, Kitson RR, Siegel D, You Q, Backos DS, Ju C, Moody CJ, Ross D (2014). 19-substituted benzoquinone ansamycin heat shock protein-90 inhibitors: biological activity and decreased off-target toxicity. Mol Pharmacol.

[38] Dikalov S, Alov P, Rangelova D (1993). Role of iron ion chelation by quinones in their reduction, OH-radical generation and lipid peroxidation. Biochem Biophys Res Commun.

[39] Dikalov SI, Rumyantseva GV, Piskunov AV, Weiner LM (1992). Role of quinone-iron(III) interaction in NADPH-dependent enzymatic generation of hydroxyl radicals. Biochemistry.

[40] Bachur NR, Gordon SL, Gee MV (1978). A general mechanism for microsomal activation of quinone anticancer agents to free radicals. Cancer Res.

[41] Monks TJ, Jones DC (2002). The metabolism and toxicity of quinones, quinonimines, quinone methides, and quinone-thioethers. Curr Drug Metab.

[42] Gutteridge JM (1984). Streptonigrin-induced deoxyribose degradation: inhibition by superoxide dismutase, hydroxyl radical scavengers and iron chelators. Biochem Pharmacol.

[43] Supko JG, Hickman RL, Grever MR, Malspeis L (1995). Preclinical pharmacologic evaluation of geldanamycin as an antitumor agent. Cancer Chemother Pharmacol.

[44] Grant S (2011). Enhancing proteotoxic stress as an anticancer strategy. Oncotarget.

[45] Schonthal AH (2013). Pharmacological targeting of endoplasmic reticulum stress signaling in cancer. Biochem Pharmacol.

[46] Hotamisligil GS (2010). Endoplasmic reticulum stress and the inflammatory basis of metabolic disease. Cell.

[47] Neznanov N, Komarov AP, Neznanova L, Stanhope-Baker P, Gudkov AV (2011). Proteotoxic stress targeted therapy (PSTT): induction of protein misfolding enhances the antitumor effect of the proteasome inhibitor bortezomib. Oncotarget.

[48] Malhotra JD, Kaufman RJ (2007). Endoplasmic reticulum stress and oxidative stress: a vicious cycle or a double-edged sword?. Antioxid Redox Signal.

[49] Schultz MA, Abdel-Mageed AB, Mondal D (2010). The nrf1 and nrf2 balance in oxidative stress regulation and androgen signaling in prostate cancer cells. Cancers (Basel).

[50] Friling RS, Bensimon A, Tichauer Y, Daniel V (1990). Xenobiotic-inducible expression of murine glutathione S-transferase Ya subunit gene is controlled by an electrophile-responsive element. Proc Natl Acad Sci U S A.

[51] Ohtsuji M, Katsuoka F, Kobayashi A, Aburatani H, Hayes JD, Yamamoto M (2008). Nrf1 and Nrf2 play distinct roles in activation of antioxidant response element-dependent genes. J Biol Chem.

[52] Blank V (2008). Small Maf proteins in mammalian gene control: mere dimerization partners or dynamic transcriptional regulators?. J Mol Biol.

[53] Motohashi H, O’Connor T, Katsuoka F, Engel JD, Yamamoto M (2002). Integration and diversity of the regulatory network composed of Maf and CNC families of transcription factors. Gene.

[54] Itoh K, Tong KI, Yamamoto M (2004). Molecular mechanism activating Nrf2-Keap1 pathway in regulation of adaptive response to electrophiles. Free Radic Biol Med.

[55] Bradshaw-Pierce EL, Pitts TM, Kulikowski G, Selby H, Merz AL, Gustafson DL, Serkova NJ, Eckhardt SG, Weekes CD (2013). Utilization of quantitative *in vivo* pharmacology approaches to assess combination effects of everolimus and irinotecan in mouse xenograft models of colorectal cancer. PLoS One.

