# Supplementary material for: HSP90 inhibitor 17-DMAG exerts anticancer effects against gastric cancer cells principally by altering oxidant-antioxidant balance
Source: Oncotarget. 2017 Apr 10;8(34):56473–89. doi: 10.18632/oncotarget.17007 (PMC5593576; doi:10.18632/oncotarget.17007)
Supplement: Supplementary file 1 [file oncotarget-08-56473-s001.pdf]

HSP90 inhibitor 17-DMAG exerts anticancer effects against gastric cancer cells principally by altering oxidant-antioxidant balance

SUPPLEMENTARY MATERIALS

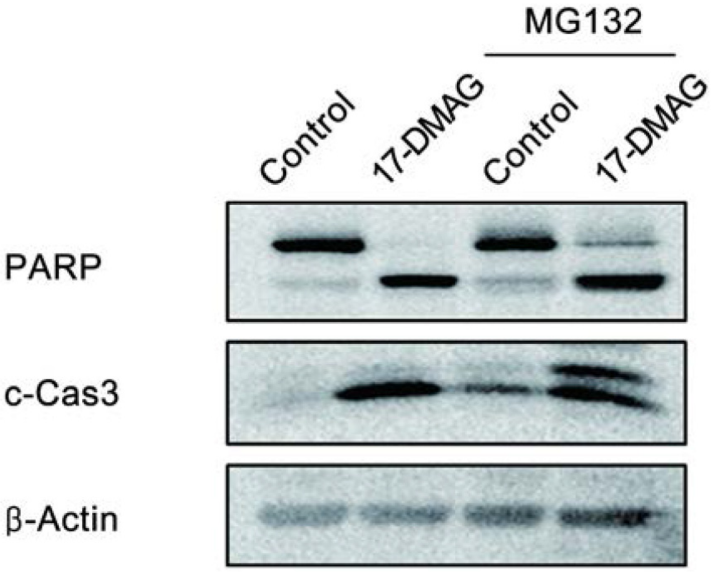

Supplementary Figure 1: Cytotoxic effect of proteasome inhibitor MG132 on the proapoptotic effect of 17-DMAG in AGS gastric cancer cells. Western blot analysis showed that MG132 promoted proapoptotic effects of 17-DMAG in both gastric cell lines. It is reasonable to think that disruption of proteasome activity causes accumulation of otherwise degradable proteins within the cell and thus constitutive ER stress causes cell growth arrest, eventually leading to cell death. Values represent means ± SD of three independent experiments. C-Cas3, cleaved caspase 3; Ct, control; PARP, polyADP-ribose polymerase.

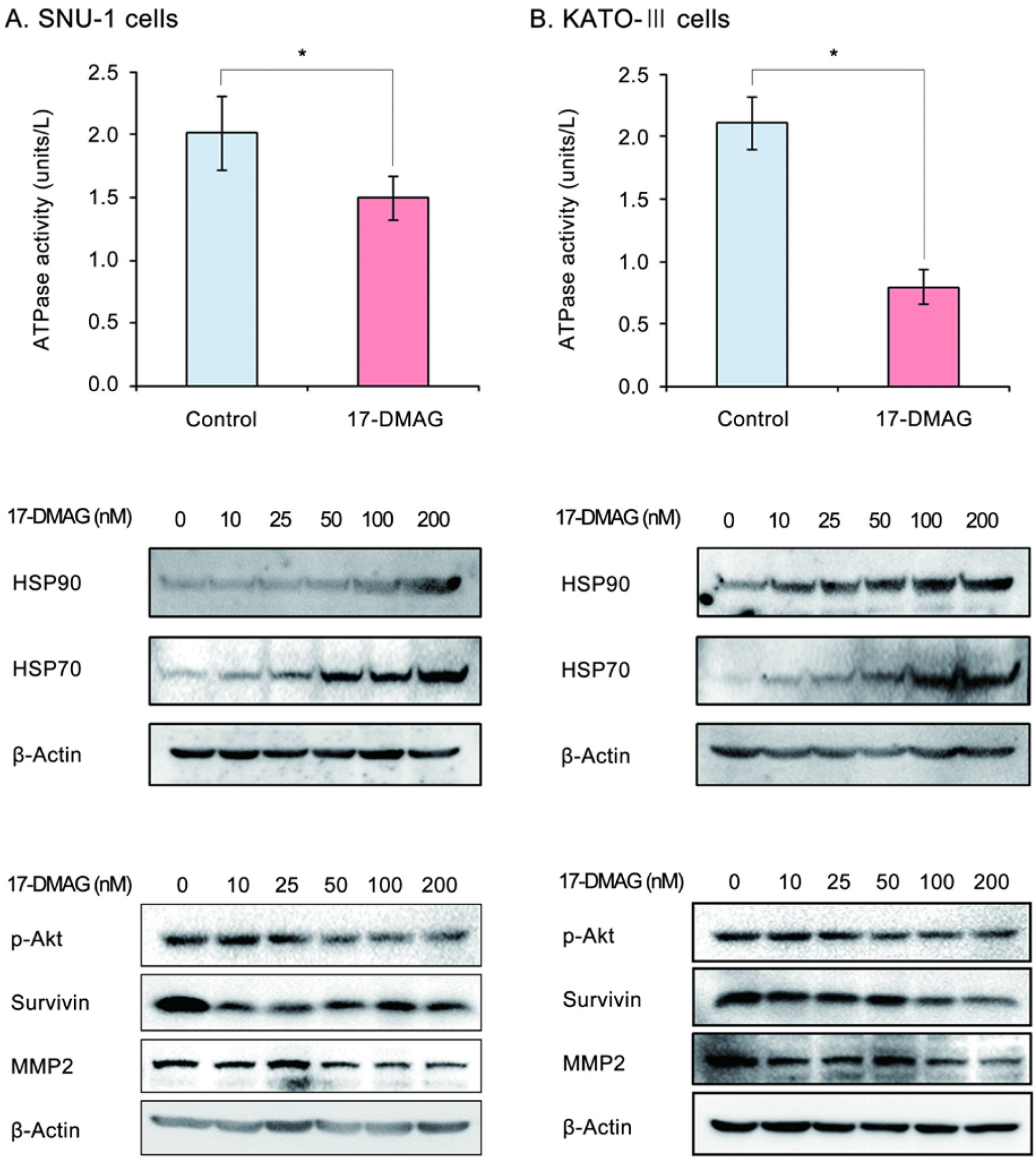

Supplementary Figure 2: Action mechanism of 17-DMAG as an HSP90 inhibitor (canonical pathway) in SNU-1 and KATO-III gastric cancer cells (A) Action mechanism of 17-DMAG as an HSP90 inhibitor in SNU-1 cells. [Top] HSP90 ATPase activity assay demonstrating the inhibition of ATPase activity of Hsc90 by 17-DMAG in SNU-1 cells. **[Middle]** Western blot analysis showing dose-dependent elevation of Hsp70 and HSP90 levels in SNU-1 cells by 17-DMAG. **[Bottom]** Western blot analysis showing the dose-dependent reduction of the expression of HSP90 client proteins, such as p-Akt, survivin, and MMP2 by 17-DMAG in SNU-1 cells. **(B)** Action mechanism of 17-DMAG as an HSP90 inhibitor in KATO-III cells. **[Top]** HSP90 ATPase activity assay demonstrating the inhibition of ATPase activity of Hsc90 by 17-DMAG in KATO-III cells. **[Middle]** Western blot analysis showing dose-dependent elevation of Hsp70 and HSP90 levels in KATO-III cells by 17-DMAG. **[Bottom]** Western blot analysis showing the dose-dependent reduction of the expression of HSP90 client proteins by 17-DMAG in KATO-III cells. Values represent means ± SD of three independent experiments. GPx, Glutathione peroxidase; HSP, heat shock protein; MMP2, Matrix metalloproteinase 2; MnSOD, Manganase superoxide dismutase. *$P < 0.05$.

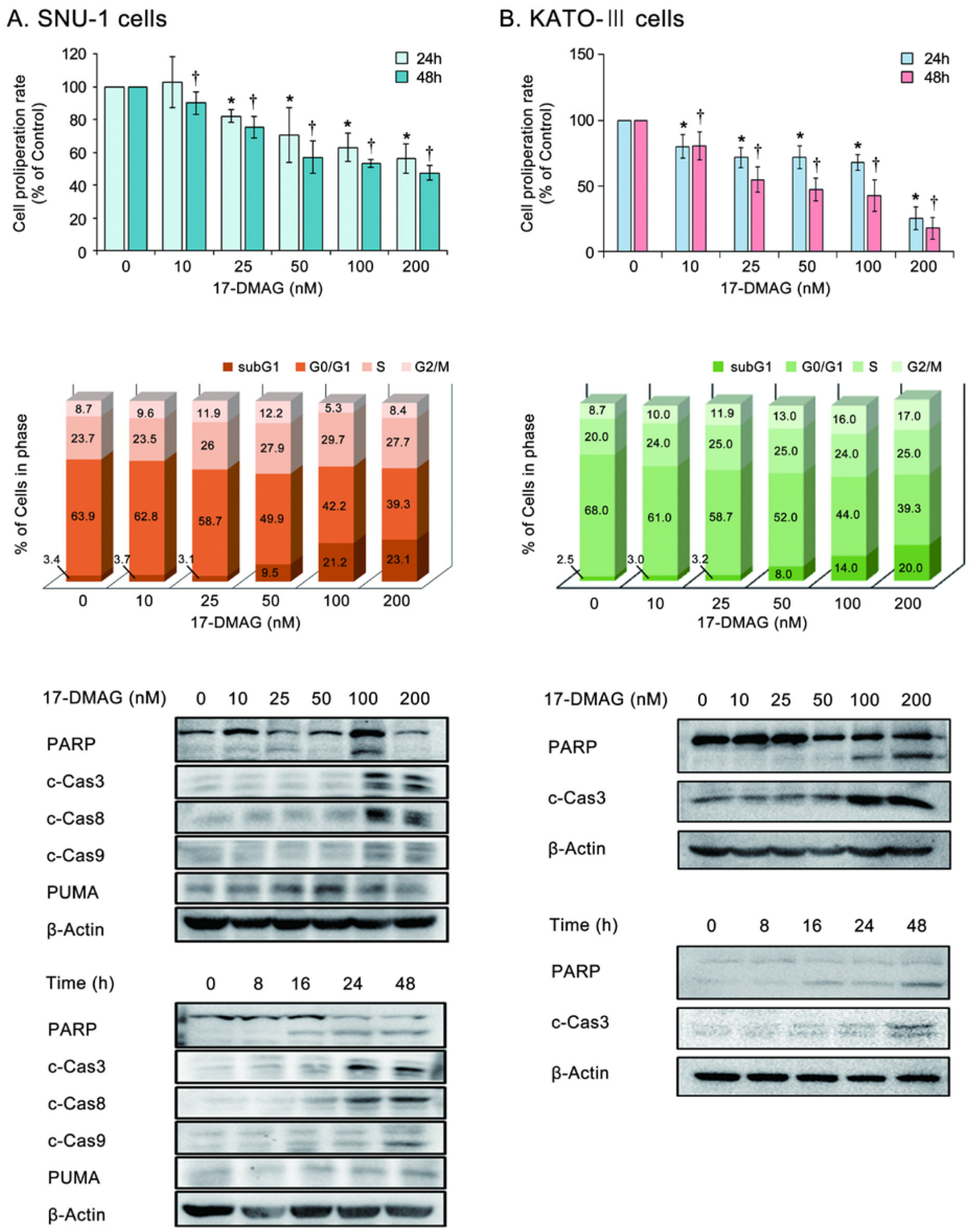

Supplementary Figure 3: 17-DMAG effects on the proliferation and apoptosis of SNU-1 and KATO-III gastric cancer cells. (A) 17-DMAG effects on the proliferation and apoptosis of SNU-1 cells. **[Top]** Proliferation assay of SNU-1 cells which were treated with graded concentrations of 17-DMAG for 24 h and 48 h. 17-DMAG resulted in significant dose- and time-dependent reduction of SNU-1 cell proliferation ($P < 0.05$). **[Middle]** 17-DMAG effects on the percentage of apoptotic cells determined by subG1 population (hypodiploid DNA). As the 17-DMAG concentration was raised, the percentage of apoptotic cells were increased, demonstrating significant proapoptotic effects of 17-DMAG. **[Bottom]** 17-DMAG effects on the expression of apoptotic proteins (PARP, c-caspase 3, c-caspase-8, c-caspase-9, and PUMA) in SNU-1 cells. Western blot analyses indicated that 17-DMAG increased the expression of apoptotic proteins of SNU-1 cells in a dose and time dependent manner. **(B)** 17-DMAG effects on the proliferation and apoptosis of KATO-III cells. **[Top]** Proliferation assay of KATO-III cells which were treated with graded concentrations of 17-DMAG for 24 h and 48 h. **[Middle]** 17-DMAG effects on the percentage of apoptotic cells determined by subG1 population. **[Bottom]** 17-DMAG effects on the expression of apoptotic proteins in KATO-III cells. in a dose and time dependent manner. Values represent means ± SD of three independent experiments. C-Cas3, cleaved Caspase 3; PARP, polyADP-ribose polymerase; PUMA, p53 Up-regulated Modulator of Apoptosis. *$P < 0.05$, † $P < 0.05$.

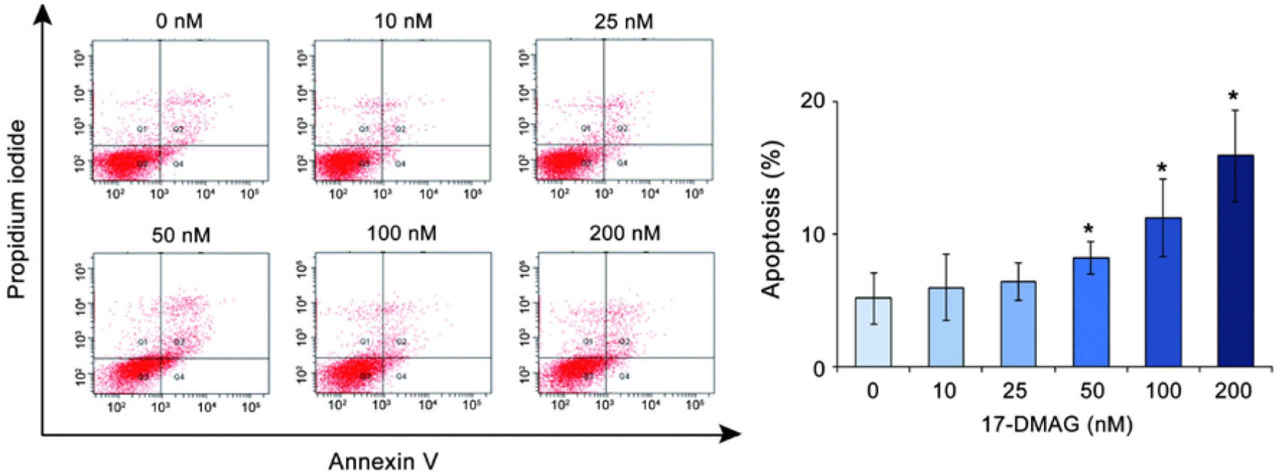

Supplementary Figure 4: Quantitative analysis of apoptosis using Annexin V/propidium iodide staining and flow cytometry in SNU-1 gastric cancer cells. **[Left]** Apoptotic cell proportion is expressed as the total percentage of Annexin V-positive cells (early and late apoptotic cells). **[Right]** Relative percentages of apoptotic cells according to varying concentrations of 17-DMAG. The number of Annexin V-positive cells was proportional to the concentration of 17-DMAG ($P < 0.05$). Values represent means ± SD of three independent experiments. *$P < 0.05$.

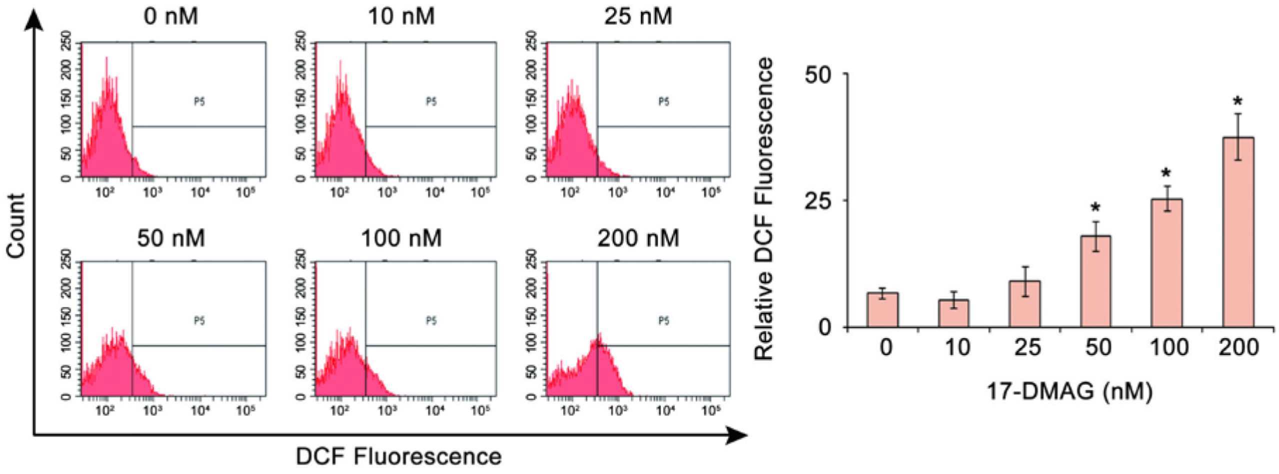

Supplementary Figure 5: 17-DMAG effects on the ROS in SNU-1 gastric cancer cells. Graphs showing the percentage of DCF fluorescent cells according to the increasing concentration of 17-DMAG. DCF-fluorescent intensity is proportional to intracellular ROS levels. 17-DMAG increased the DCF-fluorescence intensity of SNU-1 cells in a dose-dependent manner ($P < 0.05$). Values represent means ± SD of three independent experiments. DCF, 2’,7’-dichlorofluorescein. * $P < 0.05$.

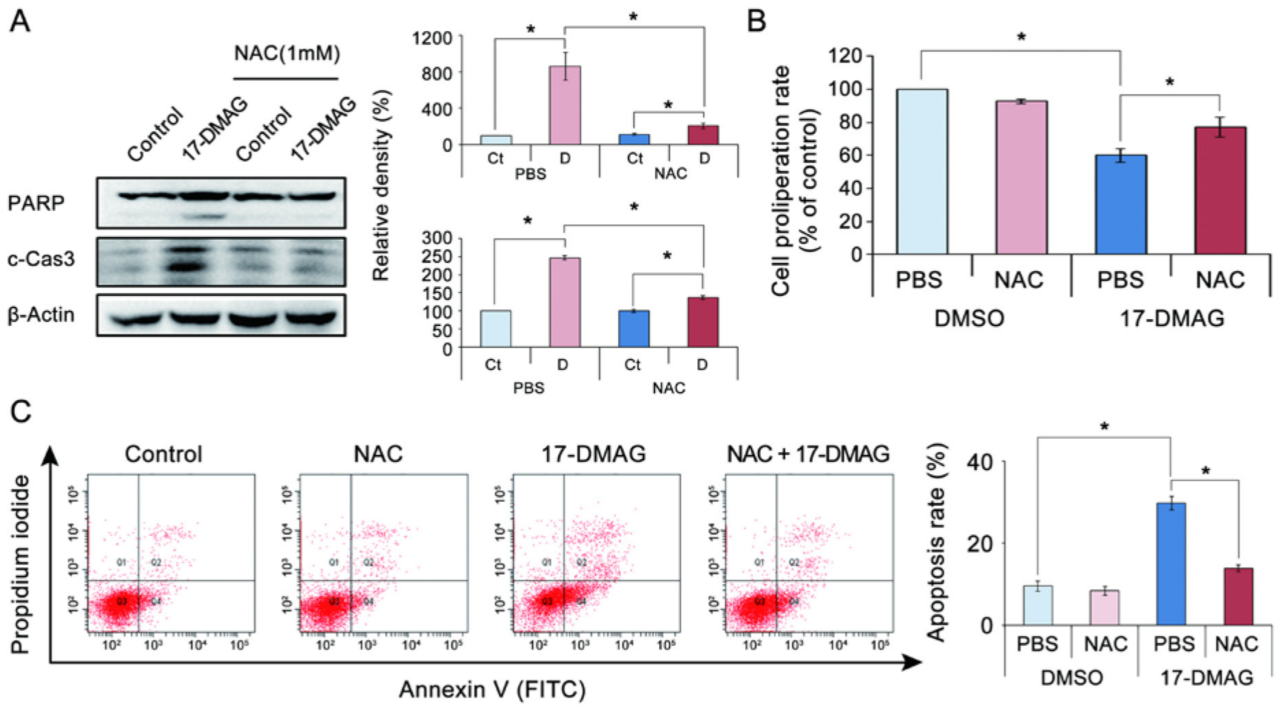

Supplementary Figure 6: Identification of action mechanism of 17-DMAG with relation to ROS in SNU-1 gastric cancer cells. (A) Effects of ROS inhibitor N-acetyl-L-cysteine (NAC) on the expression of apoptotic proteins of SNU-1 cells with or without treatment with 17-DMAG. 17-DMAG significantly increased the expression of apoptotic proteins (PARP and c-Cas3) ($P < 0.05$), which was decreased by the addition of ROS inhibitor NAC. **(B)** Effects of NAC on the proliferation of SNU-1 cells with or without treatment with 17-DMAG. 17-DMAG significantly increased the proliferation of SNU-1 cells ($P < 0.05$), which was decreased by the addition of NAC. **(C)** Effects of NAC on the apoptosis of SNU-1 cells with or without treatment with 17-DMAG. Apoptotic cells were quantified by counting the total percentage of Annexin V-positive cells. 17-DMAG significantly increased the number of Annexin V-positive cells (early and late apoptotic cells) ($P < 0.05$), which was decreased by the addition of NAC. Values represent means ± SD of three independent experiments. Ct, control; DMSO, dimethyl sulfoxide; N, 17-DMAG; NAC, N-acetyl-L-cysteine. * $P < 0.05$.

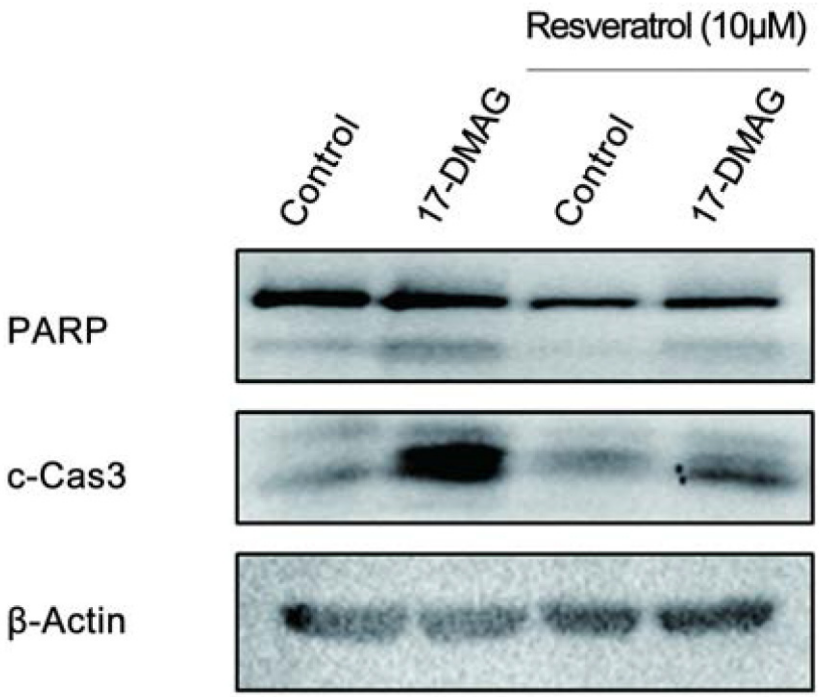

Supplementary Figure 7: Effects of the ROS inhibitor resveratrol on the expression of apoptotic proteins of AGS cells treated with 17-DMAG. Resveratrol is a potent ROS scavenger. The results of western blot analysis indicated that the addition of resveratrol significantly reduced the expression of apoptotic proteins (PARP and c-Cas3) of which expression had been increased by 17-DMAG. Values represent means ± SD of three independent experiments. C-Cas3, cleaved caspase 3; PARP, polyADP-ribose polymerase.

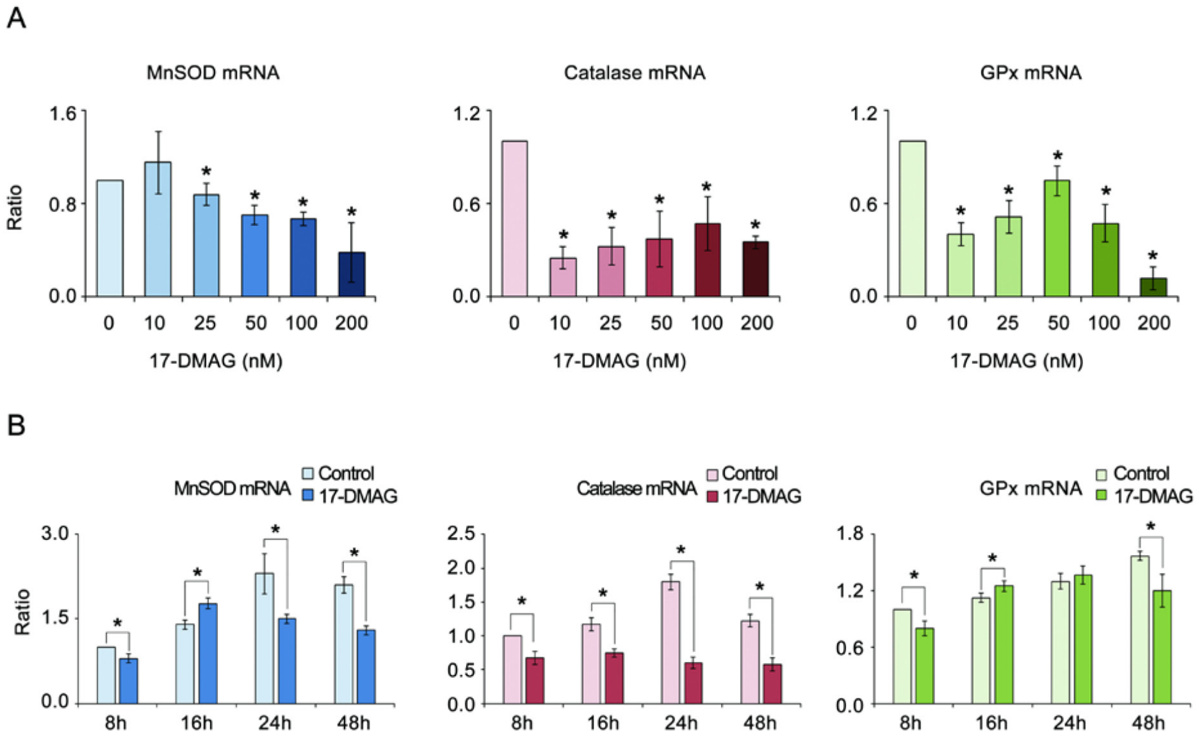

Supplementary Figure 8: Real-time PCR experiment showing 17-DMAG effects on the mRNA expression of antioxidant enzymes in AGS cells Concerning dose-dependent effects of 17-DMAG **(A)** AGS cells were treated with various concentration of 17-DMAG for 48 h, and concerning time-dependent effects **(B)** of 17-DMAG, AGS cells were treated with various duration of 100 nM 17-DMAG. Beyond a certain dose or time, 17-DMAG tended to decrease the mRNA expression of the antioxidants both dose- and time-dependently. Values represent means ± SD of three independent experiments. GPx, Glutathione peroxidase; MnSOD, Manganase superoxide dismutase. *$P < 0.05$.

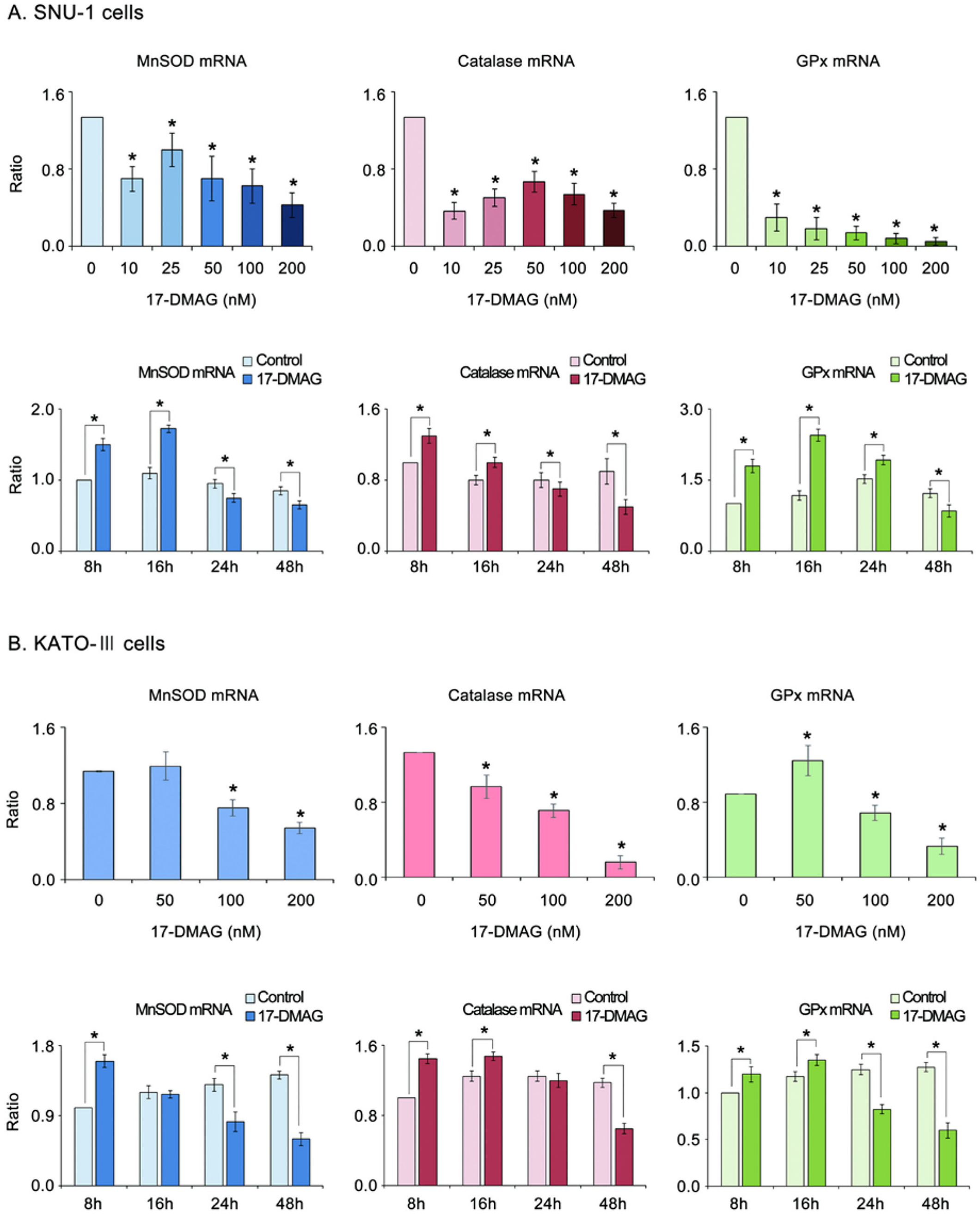

Supplementary Figure 9: Real-time PCR experiment showing 17-DMAG effects on the mRNA expression of antioxidant enzymes in SNU-1 and KATO-III cells. (A) [Top] Dose-dependent effects of 17-DMAG on the mRNA expression of antioxidant enzymes (MnSOD, catalase, and GPx) in SNU-1 cells. **[Bottom]** Time-dependent effects of 17-DMAG on the mRNA expression of antioxidant enzymes (MnSOD, catalase, and GPx). Concerning dose-dependent effects of 17-DMAG, SNU-1 cells were treated with various concentration of 17-DMAG for 48 h, and concerning time-dependent effects of 17-DMAG, SNU-1 cells were treated with various duration of 100 nM 17-DMAG. Beyond a certain dose or time, 17-DMAG tended to decrease the mRNA expression of the antioxidants both dose- and time-dependently in SNU-1 cells. **(B) [Top]** Dose-dependent effects of 17-DMAG on the mRNA expression of antioxidant enzymes in KATO-III cells. **[Bottom]** Time-dependent effects of 17-DMAG on the mRNA expression of antioxidant enzymes. Concerning dose-dependent effects of 17-DMAG, KATO-III cells were treated with various concentration of 17-DMAG for 48 h, and concerning time-dependent effects of 17-DMAG, KATO-III cells were treated with various duration of 100 nM 17-DMAG. Beyond a certain dose or time, 17-DMAG tended to decrease the mRNA expression of the antioxidants both dose- and time-dependently in KATO-III cells. Values represent means ± SD of three independent experiments. GPx, Glutathione peroxidase; MnSOD, Manganase superoxide dismutase. $^*P < 0.05$.

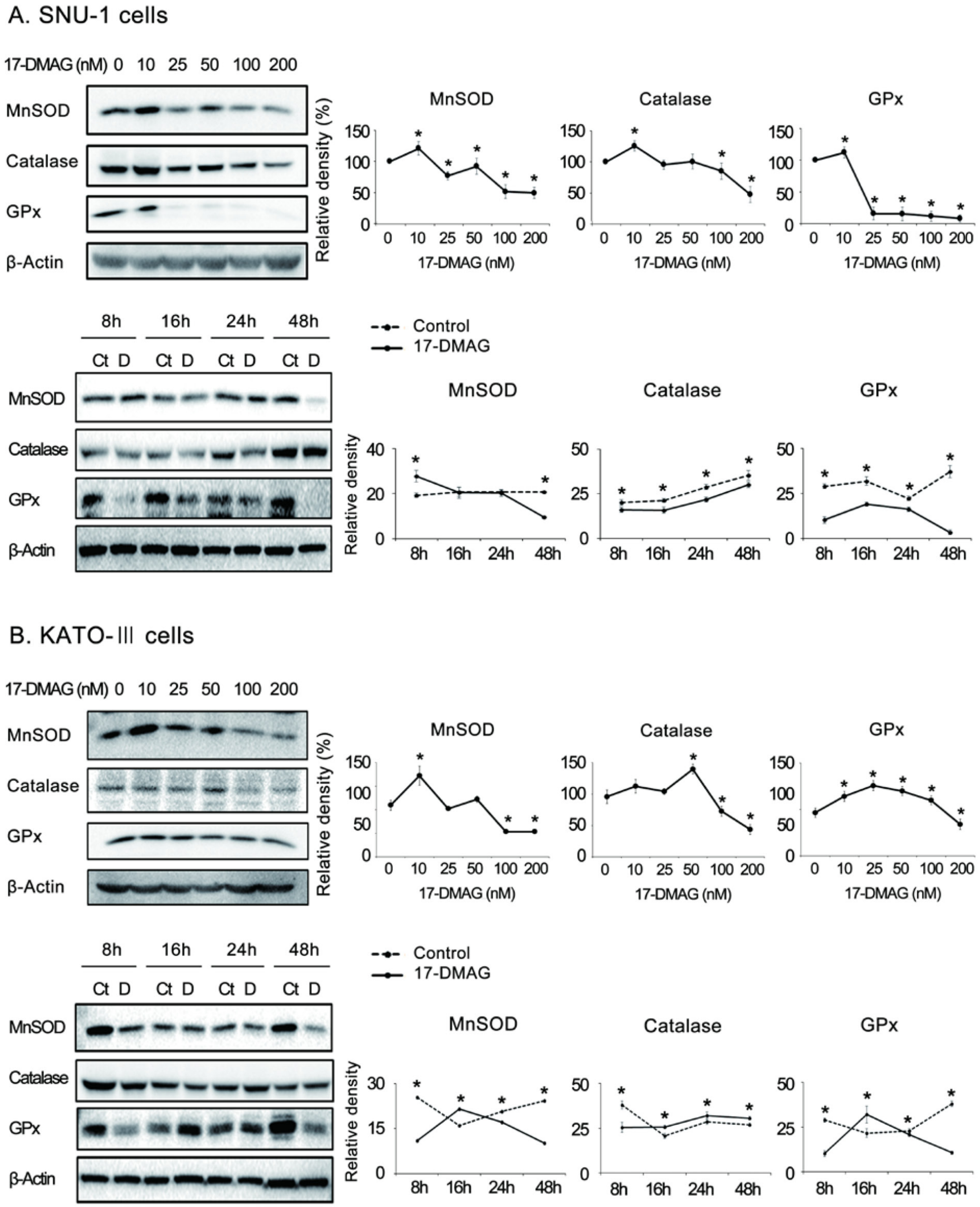

Supplementary Figure 10: 17-DMAG effects on the expression of antioxidant enzymes in SNU-1 and KATO-III gastric cancer cells. **(A)** Western blot analysis showed that 17-DMAG has the potential to reduce the expression of the antioxidants (MnSOD, catalase, and GPx) in SNU-1 cells on a dose **[Top]** and time **[Bottom]** dependent manner. **(B)** Western blot analysis showed that 17-DMAG has the potential to reduce the expression of the antioxidants (MnSOD, catalase, and GPx) in KATO-III cells on a dose **[Top]** and time **[Bottom]** dependent manner. Values represent means ± SD of three independent experiments. GPx, Glutathione peroxidase; MnSOD, Manganase superoxide dismutase. *$P < 0.05$.

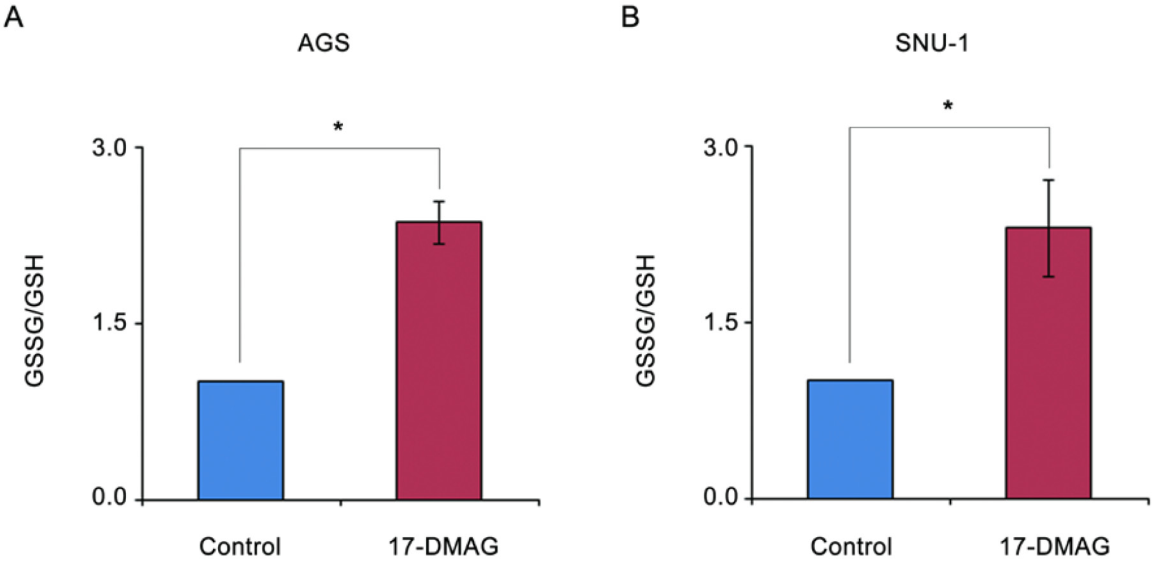

Supplementary Figure 11: Effect of 17-DMAG on the ratio of oxidized glutathione (GSSG) to reduced glutathione (GSH) in AGS **(A)** and SNU-1 **(B)** cells. Glutathione (GSH) has strong antioxidant properties, and plays an important role in protecting cells against oxidative stress. Thus, GSSG/GSH ratio is one of the useful indicator of oxidative stress. 17-DMAG induced the significantly higher GSSH/GSH ratio in both gastric cancer cell lines, demonstrating that 17-DMAG significantly decreased the intracellular glutathione levels. Values represent means ± SD of three independent experiments. Ct, control; GSH, reduced glutathione; GSSG: oxidized glutathione.

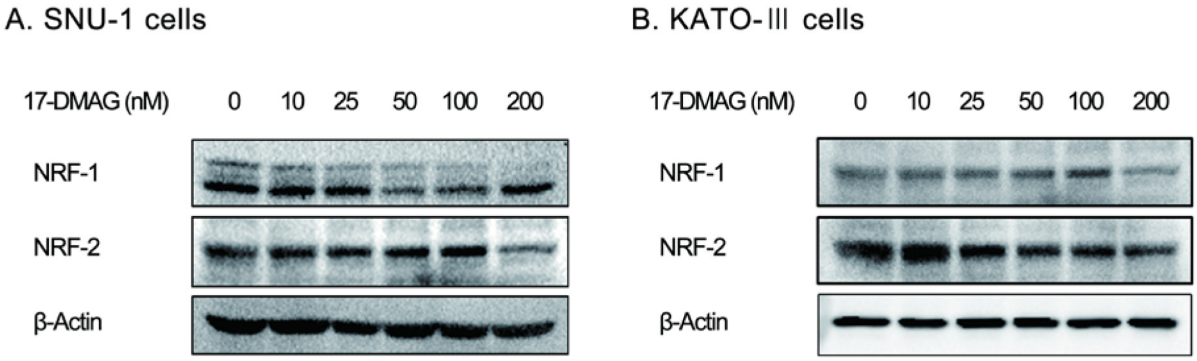

Supplementary Figure 12: 17-DMAG effects on the expression of NRFs (NRF-1 and NRF-2) in SNU-1 and KATO-III gastric cancer cells. Western blot analysis showed that the 17-DMAG potential of reducing the expression of the NRF-1 and NRF-2 dose-dependently in SNU-1 **(A)** and KATO-III **(B)** cells. Values represent means ± SD of three independent experiments. NRF-1, nuclear respiration factor; NRF-2, nuclear respiration factor-2. *$P < 0.05$.

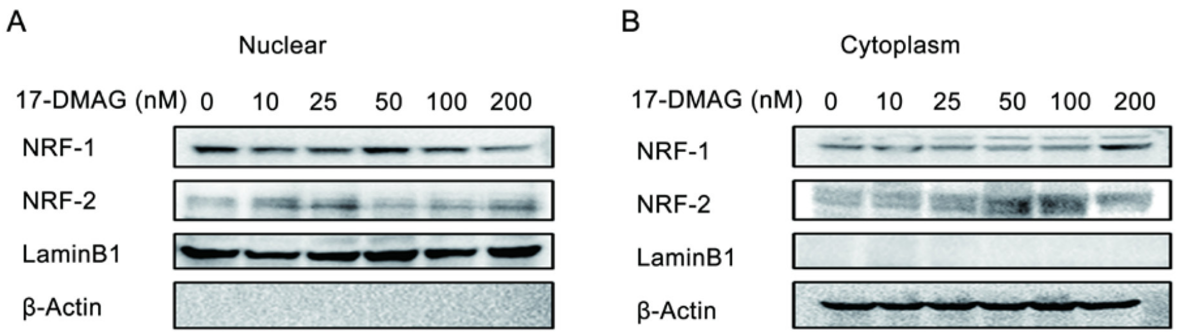

Supplementary Figure 13: 17-DMAG effects on the translocation across nuclear membrane of NRFs in AGS gastric cancer cells. Nuclear and cytoplasmic western blot analyses were performed separately. Successful separation of the nucleus and cytoplasm was verified by the detection of lamin B1 (a nuclear marker) exclusively in western blots of nuclear fractions. **(A)** Western blot analysis showing the expression of NRFs in the nuclei of AGS cells according to the concentration of 17-DMAG. **(B)** Western blot analysis showing the expression of NRFs in the cytoplasm of AGS cells according to the concentration of 17-DMAG. Values represent means ± SD of three independent experiments. NRF-1, nuclear respiration factor; NRF-2, nuclear respiration factor-2. $^*P < 0.05$.

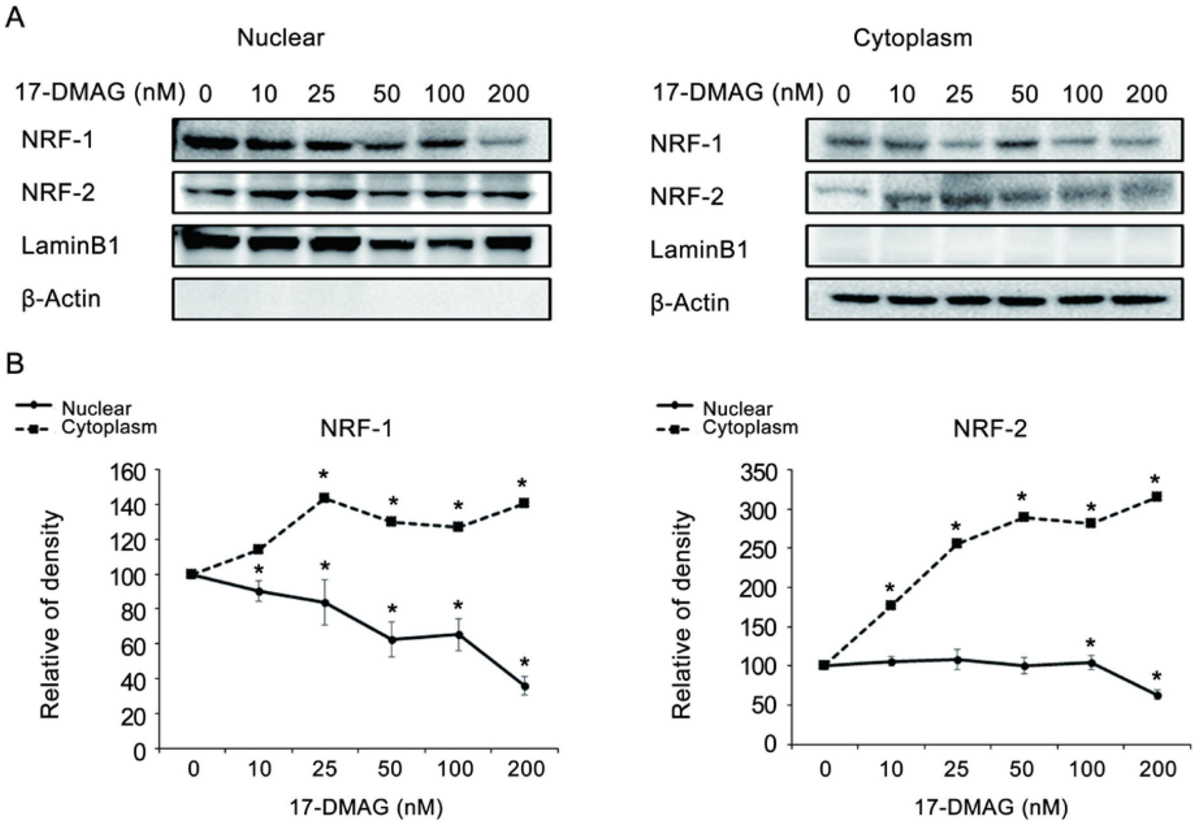

Supplementary Figure 14: 17-DMAG effects on the translocation across nuclear membrane of NRFs in SNU-1 gastric cancer cells. (A) [Left] Western blot analysis showing the expression of NRFs in the nuclei of SNU-1 cells according to the concentration of 17-DMAG. **[Right]** Western blot analysis showing the expression of NRFs in the cytoplasm of SNU-1 cells according to the concentration of 17-DMAG. **(B)** The graph showing the relative densities of the bands of western blot analysis which was performed after separation of cytoplasmic and nucleic fractions of SNU-1 cells. The expression of NRF-1**[Left]** and NRF-2 **[Right]** was dose-dependently decreased in the nuclei and increased in the cytoplasm after treatment with 17-DMAG. Values represent means ± SD of three independent experiments. NRF-1, nuclear respiration factor; NRF-2, nuclear respiration factor-2. *$P < 0.05$.
